# *Drosophila* FoxP Mutants Are Deficient in Operant Self-Learning

**DOI:** 10.1371/journal.pone.0100648

**Published:** 2014-06-25

**Authors:** Ezequiel Mendoza, Julien Colomb, Jürgen Rybak, Hans-Joachim Pflüger, Troy Zars, Constance Scharff, Björn Brembs

**Affiliations:** 1 Inst. Biol. – Behavioral Biology, Freie Universität Berlin, Berlin, Germany; 2 Inst. Biol. – Neurobiology, Freie Universität Berlin, Berlin, Germany; 3 Neuroethology, Max-Planck Institute for Chemical Ecology, Jena, Germany; 4 Biological Sciences, University of Missouri, Columbia, Missouri, United States of America; 5 Institut für Zoologie - Neurogenetik, Universität Regensburg, Regensburg, Germany; Tohoku University, Japan

## Abstract

Intact function of the Forkhead Box P2 (FOXP2) gene is necessary for normal development of speech and language. This important role has recently been extended, first to other forms of vocal learning in animals and then also to other forms of motor learning. The homology in structure and in function among the FoxP gene members raises the possibility that the ancestral FoxP gene may have evolved as a crucial component of the neural circuitry mediating motor learning. Here we report that genetic manipulations of the single *Drosophila* orthologue, *dFoxP*, disrupt operant self-learning, a form of motor learning sharing several conceptually analogous features with language acquisition. Structural alterations of the *dFoxP* locus uncovered the role of *dFoxP* in operant self-learning and habit formation, as well as the dispensability of *dFoxP* for operant world-learning, in which no motor learning occurs. These manipulations also led to subtle alterations in the brain anatomy, including a reduced volume of the optic glomeruli. RNAi-mediated interference with *dFoxP* expression levels copied the behavioral phenotype of the mutant flies, even in the absence of mRNA degradation. Our results provide evidence that motor learning and language acquisition share a common ancestral trait still present in extant invertebrates, manifest in operant self-learning. This ‘deep’ homology probably traces back to before the split between vertebrate and invertebrate animals.

## Introduction

The Forkhead Box P2 (FOXP2) transcription factor is the first gene discovered to be involved in the development of speech and language [1], [2]. The gene reveals signs of recent selection in the hominin lineage [2], [3] and natural variation in the FOXP2 gene has been found to alter grey matter concentrations in patients with schizophrenia [4]. In fact, different polymorphisms have been found to dissociate between autism spectrum disorders on one side and schizophrenia on the other [5]–[9](but see also [10]). The FoxP2 gene sequence is highly conserved in vertebrates, its expression is largely concordant and numerous experiments indicate that FoxP2 is important for modulating the neural circuits involved in vocal learning [11]–[14]. For instance, RNAi-mediated knock-down of FoxP2 gene expression in zebra finch area X, a basal ganglia structure necessary for song learning, results in an incomplete and inaccurate imitation of tutor song [13]. Recent reports broaden the functional role of FoxP genes among vertebrates to also cover other forms of motor learning and other paralogues, particularly FoxP1 [11], [12], [15]–[22]. These findings together suggest a role of FoxP genes primarily in the acquisition of skilled coordination of the movements that underlie effective vocal communication and other movements (‘motor learning hypothesis’)[11]–[13], [16]–[19], [23]–[26]. Both paralogues' importance for motor learning indicates that the single ancestral gene may once have provided this function alone.

**Figure 1:**
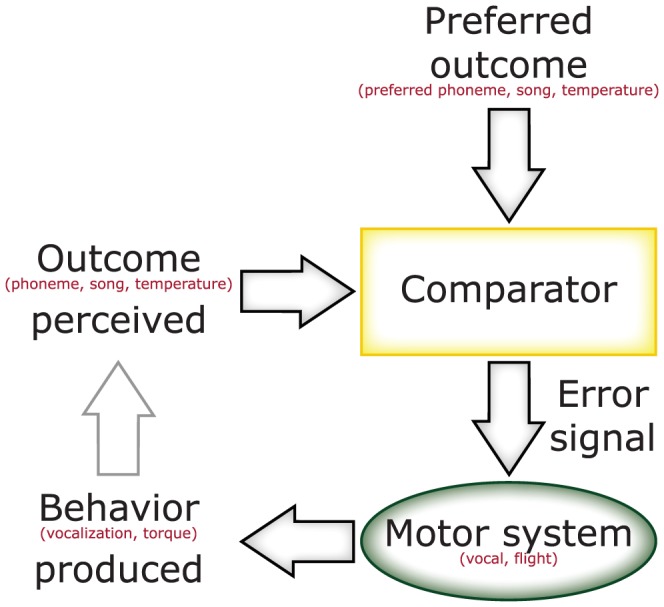
Conceptual architecture of operant feedback loops. Given the operant nature of the learning procedure, vocal learning in songbirds and humans share some conceptual aspects with operant self-learning in *Drosophila* at the torque meter. The motor system (the vocal system in songbirds and humans, the flight system in flies) generates behavioral actions (vocalizations or torque) which lead to sensory feedback (phonemes, song or heat). This actual outcome is then evaluated with respect to the preferred outcome (intended phoneme, tutor song template or preferred temperature). Any deviation from the preferred outcome will lead to a teaching signal instructing the motor system to modify the generated behavior until the desired state of the animal is reached. This schematic was modified from [30] and inspired by [27], [31], [100].

Learning to produce vocalizations by imitation is a form of motor learning that proceeds slowly from ‘babbling’ (in humans) and ‘subsong’ (in birds) towards full-fledged language and ‘crystallized’ song. This type of motor learning has been classified as a form of operant learning [27]–[29]. That is, first exploratory, highly variable actions are initiated (i.e., babbling or subsong) and then sensory feedback shapes the initiation of future behavior, reducing its variability (i.e., crystallized song, speech) [14], [30]. A particular variant of operant learning in tethered flying *Drosophila* [31], [32] parallels some features of vocal learning: the animal first initiates highly variable, exploratory actions (i.e., turning maneuvers to the left and to the right), then sensory feedback shapes the initiation of future behavior, reducing its variability (i.e., turning maneuvers only to one side, see Fig. 1). We tested the hypothesis that operant learning may share homologous aspects with vocal learning using the *dFoxP* gene. Our evidence suggests that the *Drosophila* FoxP2 orthologue, *dFoxP*, plays a critical role in operant self-learning, a form of motor learning that shares conceptually analogous features with vocal learning.

## Materials and Methods

### Fly strains

All flies were kept on standard cornmeal/molasses medium [33] at 25°C and 60% humidity with a 12 hr light/12 hr dark regime [34]. FlyBase (http://flybase.org) insertion lines (P{RS5}FoxP^5-SZ-3955^, FlyBaseID: FBti0030257; PBac{WH}FoxP^f03746^, FlyBaseID: FBti0051415; PBac{PB}c03619, FlyBaseID: FBti0044630) were crossed into a wild type *Canton S* (CS) genetic background for at least six generations. Both the CS control strain and the P{RS5}FoxP^5-SZ-3955^ insertion line were crossed over the deficiency Df(3R)ED5438, FlyBaseID: FBab0036674. We used the P [GAL4] technique [35] to express an RNAi construct from the Vienna VDRC stock center under control of the elav pan-neural GAL4 driver. We took advantage of the fact that prior to Santos *et al*. [36], exon eight of the FoxP gene was listed as a separate gene in FlyBase (CG32937) while the other exons were listed as CG16899 (now CG43067). There currently is one construct directed against CG32937 (P{GD15847}v50200, FlyBaseID: FBti0085940) which we used here to specifically target *dFoxP* isoform B. All crosses were performed reciprocally.

### mRNA extraction

Around 50 heads of the different fly strains were placed separately in 1.5 ml Eppendorf tubes, frozen in liquid nitrogen and stored at −80°C until mRNA extraction. Tissue was homogenized in 200 µl of TRIZOL reagent (Invitrogen) using a pellet pestle tip attached to a pellet pestle motor (Sigma Z359947 – 100EA; Z359971 – 1EA) and mRNA extraction was performed according to the manufacturer's instructions. Residual DNA was then degraded using Turbo DNA – free kit (Ambion) following the manufacturer's instructions. mRNA yield was determined by UV spectroscopy at 260/280 nm with Nanodrop (PEQLab Nanodrop, Spectrophotometer ND-1000). For cDNA synthesis, the same quantities of mRNA (1200 ng) were taken and Super Script III Reverse Transcriptase (Invitrogen, 18080-093) using Random Hexameres primers were used for first strand synthesis following the manufacturer's instructions. As a negative control a minus Reverse transcriptase reaction was also run with the same quantity of mRNA. For QPCR the cDNA was diluted 1∶10 in Nuclease free water (Ambion, AM9932).

### Cloning of dFoxP isoforms and PCR

Primers where designed using the software Primer3 0.4.0 (http://frodo.wi.mit.edu/). The first set of primers was designed as follows: FoxPIsoAfor (5′- AGTATTCCGAGGATGCCAAG-3′) and FoxPIsoArev (5′- CAAAACGGAAGGAGTTTGGA-3′), set on the gene CG16899, which amplified a 1347 bp product of Isoform A (NCBI acc. # JN160729) and a 1718 bp product of an intron-retention isoform (NCBI acc. # JN160730) of the FoxP gene. For sequencing exon 8 in the different strains, which is the one that changes between Isoform A and Isoform B of the FoxP gene of *Drosophila* (designated as CG32937 in the FlyBase), we used: FoxPIsoBfor (5′- AAGAATGCGATTCGTACGAAC-3′) and FoxPIsoBrev (5′-TATAATTTCCGAATCCGAACC-3′) which amplified a 532 bp fragment of the last exon (NCBI acc. # JN160731 and NCBI acc. # KF192848-KF192876). For all PCRs we used 1 µl of un-diluted cDNA of each strain. We ran a gradient PCR (50–65°C) with wild type *Drosophila* cDNA to determine the optimal annealing temperature. The PCR conditions were 94°C for 5 min, denaturation at 94°C for 5 s, annealing for 30 s at 60°C for Isoform A and 56.7°C for Isoform B, elongation for 2 min for Isoform A and 40 s for Isoform B at 72°C, 35 cycles, a last elongation at 72°C for 10 minutes. With this information we designed primers to amplify the full length *FoxP* isoforms of *Drosophila melanogaster* adding a restriction site for BamHI in the forward primer followed by the start sequence and FLAG-tag at the C′-terminal part of the protein followed by a Stop codon and EcoRI restriction site. We used the same forward primer for all isoforms, since the first six exons are shared. The forward primer we used was: sFOXPdm_for (5′- GGATCCGCCACCATGCATCGGATACATGACGACGAGTATTCCGAGGATGCCAAG-3′). For isoform A we used the eFOXP899_rev (5′-GCGGAATTCCTACTTATCGTCGTCATCCTTTAATCTTTGAGACCCACATACCC -3′) which amplified a 1374 bp fragment (NCBI acc. # KF206330). For the intron retention isoform we used the eFOXP_IR_rev (5′- GCGGAATTCCTACTTATCGTCGTCATCCTTGTAATCTGTTGCATAATATATAGA-3′) which amplified a 1222 bp (NCBI acc. # KF206329). Last, we used for the isoform B the reversed primer eFOXP937_rev (5′- GCGGAATTCCTACTTATCGTCGTCATCCTTGTAATCTCGATTGTGCTCATTGGC -3′) which amplified a fragment of 1608 bp fragment (NCBI acc. # KF206331). For all PCRs we used 1 µl of un-diluted cDNA of heads of *Canton S* wild type strain. For amplification of the full Open reading frame of the FoxP isoforms we used the High Fidelity PCR Enzyme Mix (#K0192; Lot 00116896). We used 5 µl of un-diluted cDNA in 50 µl final volume. We employed a HLA- PCR with the conditions 96°C for 1 min; followed by 5 cycles of 96°C for 25 seconds, 65°C for 45 seconds and 72°C for 1minute; followed by 25 cycles of 96°C for 25 seconds, 60°C for 45 seconds and 72°C for 1 minute; followed by 6 cycles of 96°C for 25 seconds, 55°C for 1 minute and 72°C for 2 minutes and a last cycle at 72°C for 10 minutes. PCR products were examined in 0.5% EtBr agarose gels, the specific bands were cut from the gel, and purified using QIAquick Gel Extraction Kit (Qiagen, Cat. 28706). PCR products were then cut using Fast digest BamHI and EcoRI enzymes (Fermentas) and cloned into pcDNA3.1 (−) vector (Invitrogen) cut with the same enzymes. These plasmids were transformed into One Shot Top 10 *Escherichia coli* chemically competent cells (Invitrogen, C404010) and colonies with ampicilin (100 µg/ml) resistance were selected on agarose plates. Clones with the specific insert were picked and grown overnight, at 37°C in 3 ml LB/ampicilin medium in a shaker. Plasmids were purified using Invisorb Spin Plasmid Mini Two columns (Invitek, Ref 1010140300) as described by the manufacturer. Finally, the inserts were fully sequenced and analyzed.

### Sequencing of premature stop in 3955 strain

We used the FoxPIsoBfor primer (described above) and a reverse primer in the RS P element (SP1 5′-CACAACCTTTCCTCTTCAACAA-3′) to amplify a fragment of 440 bp. We sampled 2 µl of undiluted cDNA of the *FoxP^3955^* strain. The PCR conditions were 94°C for 5 min, denaturation at 94°C for 5 s, annealing for 30 s at 55°C, elongation for 1 min at 72°C, 35 cycles, and a last elongation at 72°C for 10 minutes. The PCR product was examined in 1% EtBr agarose gel, the specific band was cut from the gel, and purified using QIAquick Gel Extraction Kit (Qiagen, Cat. 28706) using the manufacturer's instructions and sequenced the fragment using the FoxPIsoBfor primer (NCBI acc. # KF198510).

### Cloning of QPCR fragments and QPCR

**Figure 2:**
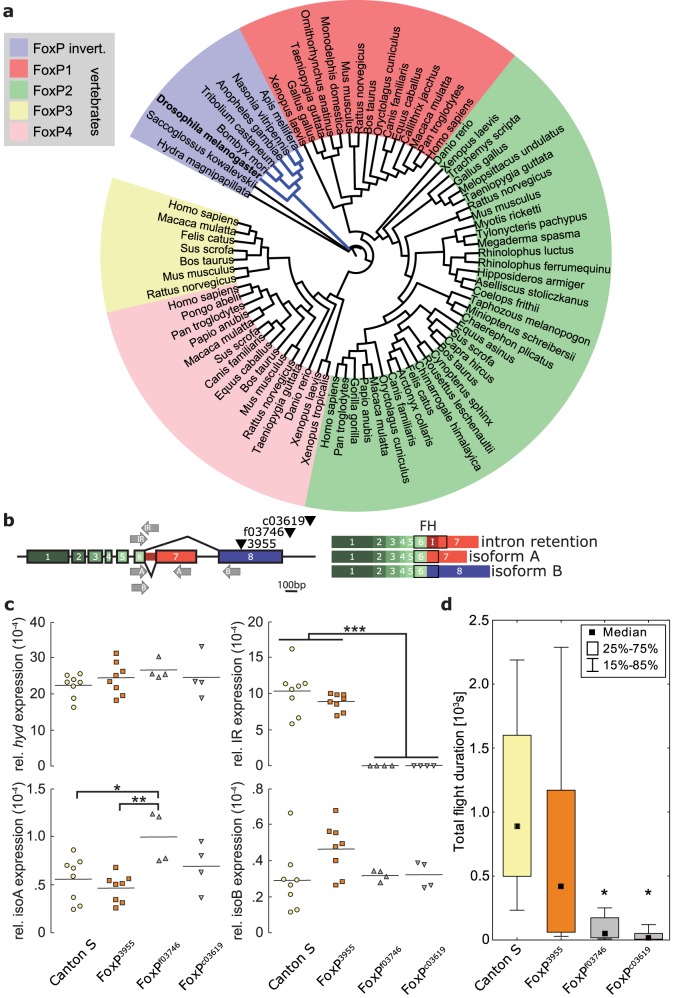
Insertion mutants of the *Drosophila FoxP* gene affect isoform expression and flight performance. **a**, Rooted phylogenetic tree using the genomic sequence of the *Drosophila FoxP* gene (see Materials and Methods). The single invertebrate FoxP gene probably corresponds to the ancestral form from which the four vertebrate genes have arisen by serial duplication. **b**, Location of the three insertions (black triangles) and qRT-PCR primer pairs (grey arrows) on the genomic structure of the *dFoxP* gene (left). Structure of the three cloned transcripts (right). IR: intron retention; FH: Forkhead-Box Domain. **c**, Expression levels of the three *dFoxP* isoform mRNAs in heads of Canton S wildtype flies and of the three insertion lines using qRT-PCR. Horizontal lines denote averages, individual circles, trinagles and squares constitute different biological replicates. * - p<0.05, ** - p<0.01, *** - p<0.001. **d**, Flight performance of Canton S wildtype flies and the three insertion lines. Asterisk denotes statistical significance compared to Canton S control flies (Kruskal-Wallis ANOVA: H(3, N = 119) = 46.02 p<0.0001, 3955: R = 77.0, p<0.4; f03746: R = 50.9, p<0.00004; c03619: R = 37.0, p<0.00001). Number of animals: 18–37. For full flight performance data see Fig. S1.

We designed QPCR primers specifically to distinguish between the different *FoxP* isoforms of *Drosophila melanogaster* (see Fig. 2b for the position of the primers in the gene) and the *hyperplastic discs* gene (*hyd*, CG9484). Primers where designed using the software Primer3 0.4.0 (http://frodo.wi.mit.edu/). Primers for *hyd* covered an intron-exon boundary, the intron retention FoxP isoform spans from exon 6 to the intron between exon 6 and 7, isoform A and B forward primer was set between exon 6 and 7 (isoform A) and exon 6 and 8 (isoform B). The settings in Primer3 were: melting temperatures between 58°C and 62°C (ΔTm<1°C), GC content between 40 and 60% and amplicon length between 90 and 120 base pairs. The size, specificity and annealing temperature was tested in a gradient PCR and checked with gel electrophoresis. The PCR conditions were 94°C for 5 min, denaturation at 94°C for 5 s, annealing for 30 s at 55–65°C, elongation for 30 s at 72°C, 35 cycles, and a last elongation at 72°C for 10 minutes. PCR products were examined in 2% EtBr agarose gels, the specific bands were cut from the gel, and purified using QIAquick Gel Extraction Kit (Qiagen, Cat. 28706). PCR products were then cloned into pGEMTeasy plasmid (Promega, Cat. A1360). These plasmids were transformed into One Shot Top 10 *Escherichia coli* chemically competent cells (Invitrogen, C404010) and colonies with ampicilin (100 µg/ml) resistance were selected on agarose plates. Clones with the specific insert were picked and grown overnight, at 37°C in 3 ml LB/ampicilin medium in a shaker. Plasmids were purified using Invisorb Spin Plasmid Mini Two columns (Invitek, Ref 1010140300) as described by the manufacturer. Finally, the inserts were fully sequenced and analyzed. Additionally, two different house-keeping genes, EF1 and RPL32 [37] were used to normalize the data. The different sets of primers used are: EMIsoA3for (5′-ACGCAGCTACGTGGAAGAAC-3′) and EMIsoA3rev (5′-TCATCGACAGTCCAAACTGC-3′) for amplification of 100 bp spanning the fragment from position 1103-1202 bp of the start codon of isoform A of FoxP between exon 6 and 7 (NCBI acc. # KF198509); EMIsoBqpcrfor (5′-GGTTCCAAAACACATTTTGCT-3′) and IRqPCR2rev (5′-GATAATATGGAGGAAAGAAGATTTACA-3′) which amplify a product of 94 bp, from 1070–1163 bp of the ORF of Intron retention isoform of FoxP from exon 6 to the intron between exon 6 and exon 7 (NCBI acc. # KF198508); IsoBqPCRfor (5′-TACGTGGAAGAATGCGATTC-3′) and EMIsoB10rev (5′-CATTATCGTCGACCATCCAA-3′) which amplify a 98bp fragment, from 1110–1207 bp of the ORF of isoform B of FoxP, between exon 6 and exon 8 (NCBI acc. # KF198507); HydQP2for (5′-ACGACGCTGGATAAGCAAAG-3′) and HydQP3rev (5′- AGATATCCAAATGGGGGACA-3′) which amplify a 97 bp fragment, from 951–1047 bp of the ORF of *hyd* (NCBI acc. # KF198506); and EF1 and RPL32 primers published in [37].

For the QPCR analysis we used an Mx3005P system and the MxPRO QPCR program (Stratagene; Agilent Technologies, U.S.A.). Every sample was run in triplicate in a 96-well plate in a total volume of 25 µl. The mixture contained 12.5 µl of Kapa SYBR Fast Universal mix (Cat. No 07-KK4600-01 Code KM4100), 0.5 µl of Kapa SYBR Fast Rox Low ((50x) Code KD 4601), 5 µl of 1∶10 diluted cDNA, 450 nM of each primer and 4.5 µl Nuclease Free water. The QPCR conditions were: 95°C for 10 minutes, followed by 40 cycles of 95°C for 30 seconds, annealing/elongation at 60°C (for intron retention, isoform B, *hyd* and RPL32), 61°C (EF1) and 65°C (isoform A) for 30 seconds. The efficiency of each gene was determined for each treatment with the slope of a linear regression model using the MxPRO QPCR program. A standard curve was generated for each gene using the cloned QPCR fragment in pGem-T easy vector and as an internal normalization between plates we used the 1×10^5^ dilution of the standard curve. We determined relative expression levels through normalization of the EF1 house keeping gene [37] which gave the best coefficient of correlation for RNAi and mutant strains (comparing EF1, RPL32 and *hyd*) using the BestKeeper software tool. Relative expression levels were determined with the comparative cycle time (Ct) method. All primers used in this study amplified the cDNA with similar efficiency (E = 100+/−8%) in a validation experiment.

ANOVA and a Tukey's Multiple Comparison post-hoc test was used to identify significant differences between strains in the *FoxP* expression with each isoform.

### Data mining of sequences of FoxP subfamily members

mRNA sequences of 80 FoxP subfamily members of invertebrates and vertebrates were downloaded from NCBI (http://www.ncbi.nlm.nih.gov/gene). We used the ORF finder from NCBI (http://www.ncbi.nlm.nih.gov/projects/gorf) to locate and cut all open reading frames.

### Phylogenetic analysis

We created alignments across all species (see Table 1 for a list) using the European Bioinformatics Institute version of ClustalW[38] (http://www.ebi.ac.uk/Tools/clustalw2). The resulting.aln file was downloaded and opened with the Bioedit program (V.7.0.9; Tom Hall, Ibis Biosciences, Carlsbad, CA92008) and saved as a.phy program for further analysis. The Phylip program (V.3.69; Joe Felsenstein, Department of Genome Sciences at the University of Washington) was used to do bootstrapping (Seqboot.exe program of Phylip) using the default parameters; Distance Matrix (dnadist.exe program of Phylip) using the default parameters; we used the neighbor joining method (neighbor.exe program Phylip) using *Hydra magnipapillata* as the outgroup and rooting the tree. To determine the consensus tree of all FoxP sequences we used the consense.exe program of Phylip changing the parameters of the *Hydra m*. outgroup and treated the tree as rooted. We used the nexus-formatted text output of the Consense program of Phylip to generate the phylogenetic tree with the online interactive tree of life (iTOL) software (http://itol.embl.de)[39].

**Table 1:** List of sequences used for the phylogenetic analysis of FoxP subfamily members.

Sequence	Accession numbers	bp
Sequence 1: FoxP_Nasonia_vitripennis	XM_001599977.1	2067 bp
Sequence 2: FoxP_Anopheles_gambiae	XM_565765.3	1098 bp
Sequence 3: FoxP_Hydra_magnipapillata	XM_002164074.1	1746 bp
Sequence 4: FoxP_Strongylocentrotus_purpuratus	DQ286749.1	1747 bp
Sequence 5: IsoA Apis	NM_001104949.1	2217 bp
Sequence 6: IsoA Tribolium	LOC657917	2706 bp
Sequence 7: IsoA Bombyxmolito	BGIBMGA004582-TA*	1239 bp
Sequence 8: FoxPIA Drosophila	NM_001104256.1	1329 bp
Sequence 9: FoxP2_Gorilla_gorilla	AF512948	2142 bp
Sequence 10: FoxP2_Homo_sapiens	NM_148898.3	2148 bp
Sequence 11: FoxP2_Mus_musculus	NM_053242.4	2145 bp
Sequence 12: FoxP2_Danio_rerio	NM_001030082.1	2094 bp
Sequence 13: FoxP2_Rattus_norvegicus	XM_002729286.1	2097 bp
Sequence 14: FoxP2_Gallus_gallus	XM_001232321.1	2127 bp
Sequence 15: FoxP2_Pan_troglodytes	NM_001009020.2	2151 bp
Sequence 16: FoxP2_Canis_familiaris	XM_860734.1	2133 bp
Sequence 17: FoxP2_Xenopus_laevis	NM_001095669.1	2121 bp
Sequence 18: FoxP2_Bos_taurus	NM_001205569.1	2130 bp
Sequence 19: FoxP2_Macaca_mulatta	NM_001033021.1	2145 bp
Sequence 20: FoxP2_Sus_scrofa	NM_001113049.1	2130 bp
Sequence 21: FoxP2_Felis_catus	NM_001113177.1	2124 bp
Sequence 22: FoxP2_Papio_anubis	NM_001168922.1	2196 bp
Sequence 23: FoxP1_Gallus_gallus	NM_001024827.1	2061 bp
Sequence 24: FoxP1_Bos_taurus	NM_001083689.1	2025 bp
Sequence 25: FoxP1_Oryctolagus_cuniculus	XM_002713313.1	1734 bp
Sequence 26: FoxP1_Callithrix_jacchus	XM_002758547.1	2034 bp
Sequence 27: FoxP1_Monodelphis_domestica	XM_001364178.1	2127 bp
Sequence 28: FoxP1_Macaca_mulatta	XM_001084998.2	2034 bp
Sequence 29: FoxP1_Canis_familiaris	XM_858603.1	2034 bp
Sequence 30: FoxP1_Pan_troglodytes	XM_001140904.1	2034 bp
Sequence 31: FoxP1_Ornithorhynchus_anatinus	XM_001509776.1	2061 bp
Sequence 32: FoxP1_Equus_caballus	XM_001498190.1	1806 bp
Sequence 33: Foxp1_Homo_sapiens	NM_032682.4	2034 bp
Sequence 34: FoxP1_Xenopus_laevis	NM_001095533.1	1824 bp
Sequence 35: FoxP1_Rattus_norvegicus	NM_001034131.1	2136 bp
Sequence 36: FoxP1_Mus_musculus	NM_001197321.1	2118 bp
Sequence 37: FoxP4_Rattus_norvegicus	NM_001108788.1	867 bp
Sequence 38: FoxP4_Canis_familiaris	XM_538914.2	1875 bp
Sequence 39: FoxP4_Macaca_mulatta	XM_001082913.2	2004 bp
Sequence 40: FoxP4_Sus_scrofa	XM_001926847.1	2064 bp
Sequence 41: FoxP4_Pan_troglodytes	XM_518463.2	2058 bp
Sequence 42: FoxP4_Papio_anubis	NM_001168744.1	2043 bp
Sequence 43: FoxP4_Bos_taurus	XM_002684495.1	2058 bp
Sequence 44: FoxP4_Pongo_abelii	XM_002816867.1	2043 bp
Sequence 45: FoxP4_Equus_caballus	XM_001501047.2	2049 bp
Sequence 46: FoxP4_X._laevis	NM_001095615.1	1926 bp
Sequence 47: FoxP4_Danio_rerio	NM_001199491.1	2091 bp
Sequence 48: FoxP4_X._tropicalis	NM_001077187.2	1938 bp
Sequence 49: FoxP4_Homo_sapiens	NM_001012426.1	2043 bp
Sequence 50: FoxP4_Mus_musculus	NM_001110824.1	2058 bp
Sequence 51: FoxP3_Mus_musculus	NM_001199347.1	1290 bp
Sequence 52: FoxP3_Homo_sapiens	NM_014009.3	1296 bp
Sequence 53: FoxP3_Rattus_norvegicus	NM_001108250.1	1290 bp
Sequence 54: FoxP1_Taeniopygia_guttata	NM_001076698.1	2052 bp
Sequence 55: FoxP4_Taeniopygia_guttata	JN160732	2007 bp
Sequence 56: FoxP2_Taeniopygia_guttata	NM_001048263.1	2136 bp
Sequence 57: FoxP3_Macaca_mulatta	NM_001032918.1	1296 bp
Sequence 58: FoxP3_Sus_scrofa	NM_001128438.1	1296 bp
Sequence 59: FoxP3_Bos_taurus	NM_001045933.1	1296 bp
Sequence 60: FoxP3_Felis_catus	NM_001083952.1	1293 bp
Sequence 61: FoxP2_Melopsittacus_undulatus	AY466101	2130 bp
Sequence 62: FoxP2_Arctonyx_collaris	EU076391.1	2121 bp
Sequence 63: FoxP2_Aselliscus_stoliczkanus	EU076392	2151 bp
Sequence 64: FoxP2_Coelops_frithii	EU076393.1	2232 bp
Sequence 65: FoxP2_Capra_hircus	EU076394.1	2199 bp
Sequence 66: FoxP2_Chimarrogale_himalayica	EU076395.1	2202 bp
Sequence 67: FoxP2_Chaerephon_plicatus	EU076396	2163 bp
Sequence 68: FoxP2_Cynopterus_sphinx	EU076397.1	2283 bp
Sequence 69: FoxP2_Equus_asinus	EU076398.1	2118 bp
Sequence 70: FoxP2_Hipposideros_armiger	EU076400	2202 bp
Sequence 71: FoxP2_Megaderma_spasma	EU076401	2123 bp
Sequence 72: FoxP2_Miniopterus_schreibersii	EU076402	2151 bp
Sequence 73: FoxP2_Myotis_ricketti	EU076403	2151 bp
Sequence 74: FoxP2_Oryctolagus_cuniculus	EU076404	2151 bp
Sequence 75: FoxP2_R.ferrumequinum	EU076405	2133 bp
Sequence 76: FoxP2_Rousettus_leschenaultii	EU076407	2262 bp
Sequence 77: FoxP2_Taphozous_melanopogon	EU076409	2154 bp
Sequence 78: FoxP2_Tylonycteris_pachypus	EU076410	2151 bp
Sequence 79: FoxP2_Trachemys_scripta	EU076411	2106 bp
Sequence 80: FoxP2_R._luctus	EU076406	2130 bp

* Bombyx mori data base.

### Stationary flight preparation

After briefly immobilizing 24–48 h old female flies by cold-anesthesia, head and thorax were glued (Sinfony Indirect Lab Composite, 3 M ESPE, St. Paul, MN, USA) to a triangle-shaped copper hook (diameter 0.05 mm) either the day before the experiment (learning tests) or 1–2 hours before the experiments (flight performance). The animals were then kept individually in small moist chambers containing a few grains of sucrose until the experiment [34].

### Flight performance measurement

Flight performance was measured as described previously [40]. The fly, glued to the hook as described above, was attached to the experimental setup via a clamp to allow stationary flight. For observation, the fly was illuminated from behind and above (Schott, 150 Watt, 15 V) and fixed in front of a polystyrene panel. Additionally, it was shielded by another polystyrene panel from the experimenter. Tarsal contact with a bead of polystyrene prevented flight initiation before the experiment started. To initiate flight, the polystyrene bead was removed and the fly gently stimulated by a puff of air. The time until the fly ceased flying was recorded (initial flight). The fly was stimulated with a puff of air each time it stopped flying. When flight was not resumed even after three consecutive stimulations, the experiment was completed and the total flight time recorded. Every stimulus after the first one, to which the fly showed a flight response, was recorded. The person scoring the flight time was unaware of the genetic identity of the animal. All animals were included in the study, including those which did not show any flight behavior (recording a flight time of zero seconds and zero flight initiations). Flight performance measurements were conducted after an initial observation during learning pilot experiments.

### Learning experiments

The core device of the set-up is the torque compensator (torque meter)[41]. It measures a fly's angular momentum around its vertical body axis, caused by intended flight maneuvers. The fly, glued to the hook as described above, was attached to the torque meter via a clamp to accomplish stationary flight in the center of a cylindrical panorama (arena, diameter 58 mm), which was homogeneously illuminated from behind. The light source was a 100 W, 12 V tungsten-iodine bulb. For green and blue illumination of the arena, the light was passed through monochromatic broad-band Kodak Wratten gelatin filters (#47 and #99, respectively) [42]. Filters were exchanged by a fast solenoid within 0.1 s. Alternatively, the arena was illuminated with ‘daylight’ by passing it through a blue-green filter (Rosco “surfblue” No. 5433). The transmission spectrum of the Rosco blue-green filter used in this study is equivalent to that of a BG18 filter (Schott, Mainz) and constitutes an intermediate between the Kodak blue and green filters [42]. An analogue to digital converter card (PCL812; Advantech Co.) fed the yaw torque signal into a computer which stored the trace (sampling frequency 20 Hz) for later analysis. Punishment was achieved by applying heat from an adjustable infrared laser (StockerYale Lasiris SNF series, LAS-SNF-XXX-830S; 825 nm, 150 mW), directed from behind and above onto the fly's head and thorax. The laser beam was pulsed (approx. 200 ms pulse width at ∼4 Hz) and its intensity reduced to assure the survival of the fly. The entire experimental procedure can be seen on video [34].

A second set-up was used by a different experimenter (JC) to first independently reproduce some of the *FoxP^3955^* and FoxP-RNAi results and then perform the habit formation experiments (see below). Instead of the torque compensator described above, we used a torque meter as described elsewhere [43], to measure yaw torque. The remaining components of the set-up were analogous to the ones described above, with a color switch (implemented using the voice coil actuator of a computer hard disk drive [44], instead of a solenoid), an analog to digital converter (ADC-USB-120FS, measurement computing Inc., 10 Commerce Way, Norton, MA 02766, USA), which transformed the analog signal into a 12 bit digital signal that feeds into the computer, and software to control the experiment and record the data (LabView, National Instruments Germany GmbH, Ganghoferstraβe 70 b, 80339 München).

Operant self-learning was performed as previously described [31], [32]. The direction for straight flight in all experiments at the torque meter was determined as the central value exactly between the maximum left and right turning yaw torque elicited by an optomotor stimulus. The fly's spontaneous yaw torque range was then divided into ‘left’ and ‘right’ domains at this value. There were no patterns on the arena wall, but the illumination was spectrally restricted by a blue-green daylight filter. During training, heat was applied whenever the fly's yaw torque was in one domain and switched off when the torque passed into the other. Punishment of yaw-torque domains was always counterbalanced. In the test phases, heat was permanently switched off and the fly's choice of yaw torque domains recorded.

The type of operant world-learning used in this study is a modification of self-learning, as described previously [45], [46]. During training, the fly was heated whenever its yaw torque passed into the domain associated with punishment, as in self-learning. In addition, whenever the fly switched yaw torque domains, not only temperature but also arena coloration were changed (from green to blue or vice versa). Thus, yaw torque domain and color served as equivalent predictors of heat. In the test phases, heat was permanently switched off and only the fly's choice of yaw torque domains/colors recorded. Punishment of the color/yaw-torque combination was always counterbalanced. It has been shown previously that self- and world-learning, despite both being operant conditioning procedures, engage different biological learning mechanisms [32], [47], [48].

Habit formation was tested also as described previously [46]: for the first 26 minutes of the experiment the color filters were present, providing sixteen minutes of training. Thereafter, the two color filters were removed from the light source and replaced by a single blue-green filter, as in the self-learning experiments. After a short, 60s familiarization training, yaw torque preference was measured with the heat permanently switched off.

The color/yaw torque domain preference of individual flies was quantified as the performance index: PI = (t_a_-t_b_)/(t_a_+t_b_). During training periods, t_b_ indicates the time the fly is exposed to the heat and t_a_ the time without heat. During tests, t_a_ and t_b_ refer to the times when the fly chose the formerly (or subsequently) unpunished or punished situation, respectively. Thus, a PI of 1 means the fly spent the entire period in the situation not associated with heat, whereas a PI of -1 indicates that the fly spent the entire period in the situation associated with heat. Accordingly, a PI of zero indicates that the fly distributed the time evenly between heated and non-heated situations and a PI of 0.5 indicates that 90 of the 120 s in that period were spent in the unpunished situation.

**Figure 3:**
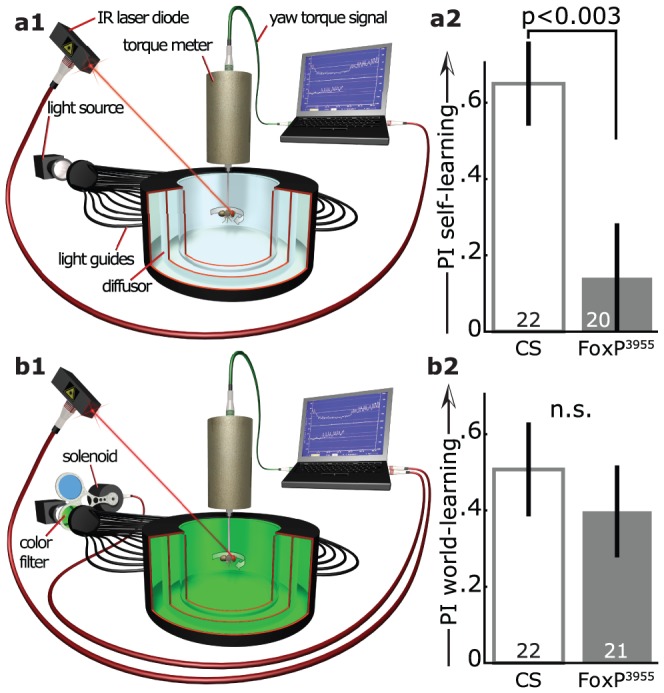
The mutant line *FoxP^3955^* was impaired in operant self- but not world-learning. **a1**, In operant self-learning the fly, tethered to a torque meter, could operate a punishing heat-beam with its yaw torque. Torque of one domain (e.g., ‘right’) may turn the heat on and the other (e.g., left) off, or vice versa. **a2**, Self-learning performance indices in a two-minute test with the heat permanently switched off immediately after eight minutes of training showed a significant impairment of *FoxP^3955^* mutant flies compared to wild type Canton S (CS) control animals (Mann-Whitney U-Test, U = 101.5, p<0.003). **b1**, In operant world-learning, the fly still operated the heat with its yaw torque, but the coloration of the environment changed with the heat as well, allowing for the colors to indicate both heat and torque domain. **b2**, World-learning performance indices in a two minute test with the heat permanently switched off immediately after eight minutes of training. There was no significant difference in performance between the two strains (Mann-Whitney U-Test, U = 201.5, p<0.5). Numbers in bars denote number of animals throughout.

**Figure 4:**
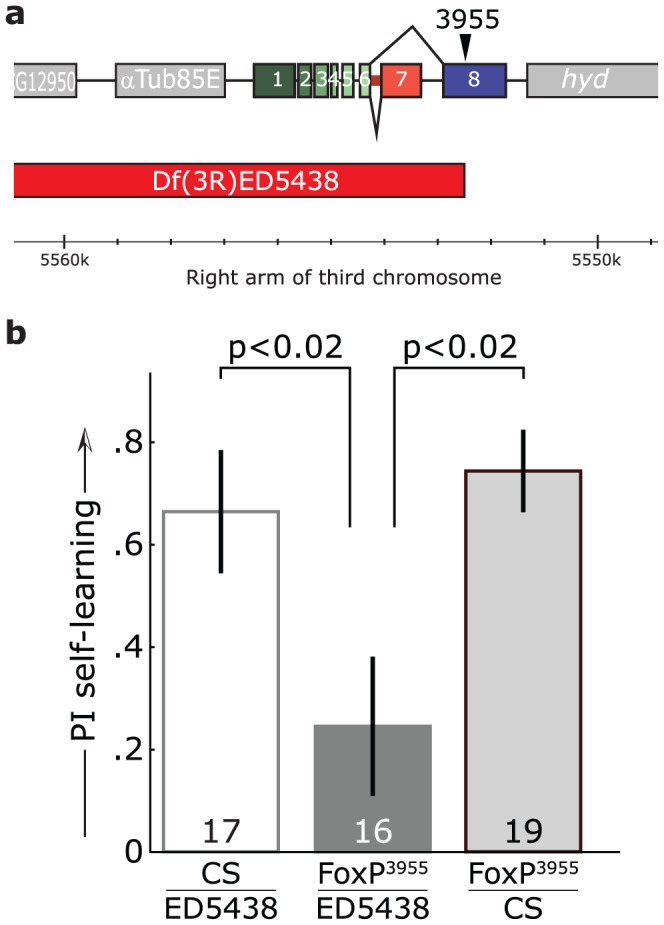
Deficiency ED5438 uncovers the *FoxP^3955^* self-learning phenotype. **a**, Genomic region of *dFoxP* gene. The deficiency deletes all exons of the *dFoxP* locus up until the 5-SZ-3955 insertion, which was used to generate the deficiency, as well as 52 upstream genes. ED5438 leaves the downstream gene *hyperplastic disks* (*hyd*) intact. **b**, Operant self-learning performance indices in a two-minute test with the heat permanently switched off immediately after eight minutes of training showed a significant impairment of *FoxP^3955^*/ED5438 flies compared to control animals in which either the deficiency or a Canton S chromosome was crossed over the 5-SZ-3955 insertion (Kruskal Wallis ANOVA, H(2, N = 52) = 10.13; p<0.007; two-sided, Bonferroni-corrected post-hoc p-values indicated in the graph).

**Figure 5:**
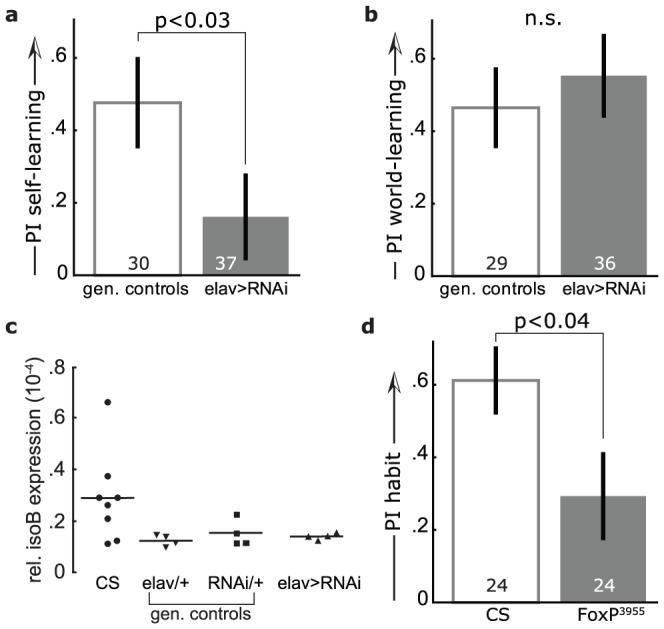
*Drosophila FoxP* full length isoform B is required for operant self-learning. **a**, Flies expressing an RNAi construct targeting the *FoxP* isoform B were impaired in operant self-learning, compared to the genetic controls (pooled, see Materials and Methods; Mann-Whitney U-Test, U = 378.5, p<0.03) in a 2-minute test immediately after eight minutes of training. See raw data or Fig. S2 for separated control groups. **b**, Both flies expressing an RNAi construct targeting the *FoxP* isoform B, as well as genetic control flies performed well in operant world-learning (Mann-Whitney U-Test, U = 420.5, P<0.2). **c**, No reduction in the expression of isoform B mRNA using qRT-PCR in flies expressing an RNAi construct targeting the *FoxP* isoform B. Canton S data are identical to those in Fig. 2c, as experiments were performed simultaneously. **d**, Mutant *FoxP^3955^* flies were impaired in habit formation. In a 2-minute self-learning test (i.e. without colors) after 16 minutes of training in world-learning (i.e. with colors), *FoxP^3955^* flies showed a significantly reduced preference for the previously unpunished turning-maneuvers, compared to wild type control animals (Mann-Whitney U-Test, U = 186.0, p<0.04). However, the mutant flies nevertheless showed a significant performance index (t-test against zero: df: 23; t = 2.54; p<0.02).

The experiments are fully automated and computer-controlled. Each fly was used only once. The time-course of each experiment was divided into consecutive periods of 2 minutes duration. Depending on whether heat was applied during such a period, it was termed a training period (heating possible) or a test period (heat off). Standard experiments consisted of two pre-test periods (labeled PI1 and PI2), four training periods (PI3, PI4, PI6 and PI7) and three memory test periods (PI5, PI8 and PI9). For experiments with extended training, the experimental time course was essentially repeated such that in total four additional training periods (PI9, PI10, PI12, PI13) followed test-PI8, as well as one additional test period (PI11). In these extended-training experiments, the color filters were removed after period 13 and period 14 was a 60 s familiarization training without colors. The final PI15 and PI16 were scored as memory tests. Depicted are always the PI's of the first two minutes after the last training period, i.e., PI8 for all standard experiments (Fig. 3, 4 and 5a, b) and PI15 for the extended-training experiments testing habit formation (Fig. 5d). All data are expressed as means ± SEM. Statistical differences between groups were tested with a Mann-Whitney U-Test or a non-parametric Kruskal-Wallis ANOVA with subsequent two-sided, Bonferroni corrected, posthoc-tests. The PIs for the genetic control strains (elav-driver and FoxP-RNAi-effector lines, respectively, crossed to Canton S) for the FoxP RNAi experiments (Fig. 5) were pooled because they did not differ in their performance and were all different from zero when tested with a t-test for single means (Fig. S2, raw data deposited at *figshare,* DOI 10.6084/m9.figshare.740444, for close scrutiny of all the training and test PIs of all groups and individual flies).

### Brain anatomy

Brains of 5–15 day old female flies were dissected and wholemounts were treated as described previously [49]. In brief, to visualize neuropil structures, immunostainings using the synaptic marker antibody nc82 (the Developmental Studies Hybridoma Bank; DSHB; this mouse monoclonal antibody binds to an epitope of the *bruchpilot* gene product [50]) were performed. Brains were fixed in 4% paraformaldehyde in phosphate-buffered solution (PBS; 0.1 M, pH 7.4) for 30 min on ice. Then the brains were washed with PBS containing 0.2% Triton X-100 (PBST) for 60 min (3 times for 20 min) at room temperature (RT). After being blocked in 5% normal goat serum (NGS) in PBST (PBST-NGS) for 60 min at RT, the brains were incubated in 1∶30 nc82 antibody in PBST-NGS for 2 days at 4°C. Then the brains were washed for 60 min at RT and incubated in 1∶200 goat anti-mouse Alexa Fluor 488 (Invitrogen) for 2 days at 4°C. Afterwards, brains were washed several times, ten minutes each, and mounted in Vectashield (Vector, Burlingame, CA) using custom-made slides and coverslips with standard 0.170 µm thickness.

Confocal images were taken with a Zeiss LSM510 confocal microscope (Carl Zeiss, Jena, Germany) using the Ar-Kr 488-nm laser line and a 40x water immersion (C-Apochromat) objective (NA: 1.2, Carl Zeiss) resulting in a voxel resolution of approximately 0.3×0.3×1 µm for a single section. In all confocal scans we used a pixel resolution of 1024×1024 in xy-axis and an 8 bit intensity resolution. The wholemount brains were thus scanned in tiled stacks, the central brain and each optic lobe separately.

Confocal image stacks were imported into the 3D software Amira (version 4.1; Visage Imaging, Berlin; San Diego, CA, USA), using the three-dimensional visualization and segmentation modules. In the case of tiled images, stacks were combined with the ‘Merge’ module. Neuropil borders were either manually segmented or semi-automatically using the 3D reconstruction module, compiled using the ‘LabelField Editor’ in the ‘Amira Image Segmentation’. The segmentation results were image stacks of type ‘LabelField’ that assign a label, which represents a distinct (brain) structure, to each voxel. The labels were rendered as surfaces for each neuropil and the ‘Statistics’ module was used for volumetric measurements.

The major neuropils of the *Drosophila* brain were defined according to the atlas proposed by Chiang et al. [51]. Only neuropils where the synaptic marker nc82 clearly defined the subneuropil borders were selected for volumetric measurements. These are 7 bilateral neuropils, among them the output areas of the optic and olfactory peripheral nervous system, i.e. optic tubercle/glomeruli and the mushroom bodies, respectively (medulla, lobula, lobula plate, mushroom bodies, optic tubercle, optic glomeruli, antennal lobes). Additionally, four non-bilateral neuropils belonging to the central complex (ellipsoid body, noduli, fan-shaped_body, protocerebral bridge) were reconstructed. The remaining neuropils of the central brain (i.e. the protocerebral lobes) were summarized as one neuropil. All values are expressed as a percentage of the sum of all brain neuropils measured.

**Figure 6:**
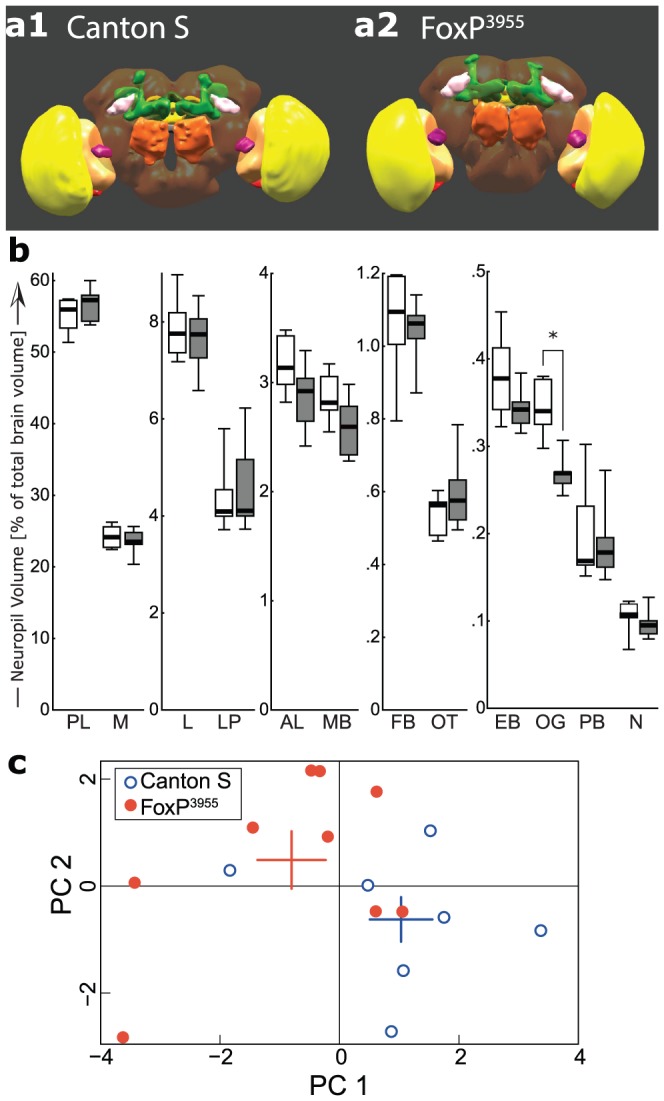
Subtle morphological alterations in the brains of FoxP^3955^ mutants. **a**, Three-dimensional surface renderings of typical fly brains from wild type Canton S (**a1**) and *FoxP^3955^* mutants (**a2**). PLOS ONE can only handle 3D PDF figures as part of the supplementary files. Hence, the 3D functionality for Figure 6 is available as Figure S3. Alternatively, a fully functional PDF will be hosted on BBs website. **b**, Quantitative volumetric analysis of eleven major neuropils (M – medulla, L – lobula, LP – lobula plate, MB – mushroom bodies, AL – antennal lobes, FB – fan-shaped body, OT – optic tubercle, EB – ellipsoid body, OG – optic glomeruli (purple in **a**), PB – protocerebral bridge, N – noduli) revealed a significant reduction in the volume of the optic glomeruli in *FoxP^3955^* flies (Mann-Whitney U-Test, U = 2.0, p<0.002). The volume of the remaining neuropils (denoted PL – protocerebral lobes) did not differ significantly. Asterisk – significant difference with a Bonferroni-corrected level of p<0.004. Black stripes – median, boxes – 25–75% percentiles, whiskers – total range. Grey boxes indicate *FoxP^3955^*, white boxes Canton S. **c**, Principal Components Analysis of the volumetric data. Plotted are the factor loadings of the individual flies on the two first components. Colored bars indicate means and standard errors (PC). Factor loadings are significantly different between Canton S and *Foxp^3955^* for PC1 (Mann-Whitney U-Test, U = 52.0, p<0.04), but fail to reach significance for PC2. Number of brains analyzed: 7 (Canton S) and 9 (*Foxp^3955^*).

Amira surface files were exported as wavefront (.obj) files. Wavefront files of neurons and neuropil surfaces were imported with the Adobe 3D Reviewer to Adobe Acrobat Pro Extended (Adobe Systems, Inc.) [52]. The images in the PDF version (Figure 6a) of this publication can be viewed by using the 3D viewer mode of the Acrobat Reader (version 9 and higher, which is freely available at http://get.adobe.com/de/reader).

The Principal Component Analysis (PCA) was performed on the symmetric correlation matrix (Pearson's method) of the volume percentage data using the R statistics package (http://r-project.org). PCA is a method that allows identifying the major patterns of correlations among multivariate data and thus condensing as much of the variation in the data as possible in few dimensions – the principal components (PC). Factor loadings for each individual fly were plotted for the first two (2D plot) or three (3D plot movie) components. Mean and standard error of the loadings were calculated for each of the three components and plotted as well. The ellipsoids in the 3D representation represent the 70% confidence interval using the covariance between the three first components.

## Results

### Flight performance and dFoxP expression in three insertion lines

The four different FoxP genes in vertebrates probably arose from serial duplications of a single ancestral FoxP gene after the separation from the invertebrate clades (Fig. 2a). The invertebrate FoxP orthologue corresponds most closely to the ancestral form of the gene at the base of the bilateria [36], thus lending itself to investigating the depth of the functional conservation among the members of the FoxP gene family. Two FoxP isoforms generated by differential splicing have been described for the fruit fly *Drosophila melanogaster* [36] and we discovered a third transcript, generated by intron retention (Fig. 2b, c). All three isoforms differ in the sequence of the Forkhead Box domain, presumably resulting in different DNA binding properties [53].

We studied *dFoxP* gene expression in three fly lines each with a distinct insertion in or near the last *dFoxP* exon, but otherwise genetically highly similar to the Canton S control strain (lines *FoxP^3955^*, *FoxP^f03746^* and *FoxP^c03619^*, Fig. 2b). We performed quantitative polymerase chain reaction following reverse transcription (qRT-PCR), to uncover the effects of the insertions on *dFoxP* gene expression. None of the insertion lines showed any deviation in the level of the neighboring gene, *hyperplastic discs* (*hyd*). In contrast, the three lines differed with respect to expression of the three *dFoxP* isoforms (Fig. 2c). We could not detect any expression of the intron retention isoform in the lines *FoxP^f03746^* and *FoxP^c03619^*. Line *FoxP^f03746^* showed elevated expression of isoform A and line *FoxP^3955^*, where the P-element had inserted into the last exon of *dFoxP*, does not show any significant difference in any of the isoforms. Genome and RNA sequencing revealed that the inserted P-element of line *FoxP^3955^* was transcribed within exon 8, resulting in a premature stop codon, such that the putative isoform B protein is truncated at position 486 (about 93% of the original size) and its final amino acid is changed to an alanine instead of aspartic acid (NCBI acc. # KF198510). Additionally, *FoxP^3955^* is the only line in which the mean isoform B expression levels appear higher than isoform A levels. In behavioral experiments measuring flight performance, tethered *FoxP^f03746^* and *FoxP^c03619^* flies flew significantly less than the Canton S control strain, whereas flying time of *FoxP^3955^* animals did not differ significantly from the control animals (Fig. 2d). Because our learning experiments required between 18-30 minutes of sustained flight, we focused our subsequent learning experiments on *FoxP^3955^*.

### Flies with manipulated dFoxP expression are specifically impaired in operant self-learning

One prediction of the hypothesis that the ancestral FoxP gene played a role in the forms of motor learning most similar to vocal learning is that manipulations of the *Drosophila FoxP* gene should affect learning with reference to its own body, ‘self-learning’ [47], [48], more than other forms of operant learning [32]. In the operant self-learning paradigm that we used [31], the tethered fly's attempts to turn are measured by a torque meter and divided into two domains, roughly corresponding to ‘left’ and ‘right’ turns, respectively (Fig. 3a1). Fixed in space in a featureless environment, the fly alternates between turning directions in a highly variable, random-like fashion [54]. Making punishment by an infrared heat beam contingent on one set of maneuvers (e.g., right turning attempts), leads to a reduction in the variability of the behavior as the fly restricts its turning maneuvers to the unpunished side (i.e., left turning attempts). This reduction outlasts the application of the heat beam after a given training period and the flies restrict their yaw torque to the previously unpunished side, even when the heat is permanently switched off [31], [32].

After eight minutes of training, wild type flies of the strain Canton S spent a significantly larger fraction of the 2-minute test without heat initiating previously unpunished turning attempts (82.5±5.2%) than *FoxP^3955^* mutant flies (57.1±6.9%; Fig. 3a2), with a structural mutation in the last exon of the *dFoxP* gene, but with wild type expression levels. To test if the flies possessed the general capabilities to solve these kinds of learning tasks, i.e., generate variable yaw torque, sense and learn to avoid the heat and restrict their yaw torque in the absence of heat, we tested *FoxP^3955^* flies in a very similar experiment, with only a single, crucial difference. Adding a solenoid with green and blue color filters to the setup allowed us to alternate the coloration of the fly's environment between the two colors together with the switch in the turning attempts. For instance, left turning attempts might have led to green coloration and right turning attempts to blue coloration or vice versa. This alteration allowed the *FoxP^3955^* mutant flies to use the colors as external cues (‘world-learning’ [47], [48]) instead of their own behavior (‘self-learning’) to predict the heat punishment, rescuing their performance (Fig. 3b). This result demonstrates that *FoxP^3955^* mutant flies are able to generate variable yaw torque, sense and learn to avoid the heat and restrict their yaw torque in the absence of heat.

The 5-SZ-3955 insertion had been introgressed into the Canton S genetic background in order to minimize the chance of genetic aberrations elsewhere potentially causing a phenotype. We used an additional strategy classically used to map a phenotype to a specific mutation. We crossed both *FoxP^3955^* mutant flies and Canton S control flies over a deficiency which deleted all exons of the *dFoxP* gene up until the 5-SZ-3955 insertion site, as well as 52 other, upstream genes (Fig. 4a). In parallel, we tested the progeny of a Canton S - *FoxP^3955^* cross for a potential dominant effect of the 5-SZ-3955 insertion. The heterozygote F1 offspring of each of the three crosses were tested in operant self-learning as described above. The recessive self-learning phenotype of the *FoxP^3955^* mutation was uncovered by the deficiency (Fig. 4b), indicating that indeed the 5-SZ-3955 insertion is the cause for the mutant phenotype of these flies. Together with the truncated isoform B expression in *FoxP^3955^*, the above results encouraged us to attempt a regulatory manipulation of *dFoxP* expression. We hypothesized that isoform B may be specifically required for operant self-learning.

To test this hypothesis, we targeted isoform B by expressing a specific RNAi construct under the control of the pan-neuronal elav-GAL4 driver line. These flies did not appear impaired in their flight ability and hence their flight performance was not quantified. Consistent with our hypothesis, subjecting flies expressing an RNAi-construct targeting isoform B to self- and world-learning yielded a phenocopy of the mutant data (Fig. 5a, b). We assessed the effectiveness of the RNAi method using qRT-PCR, but the level of isoform B mRNA was unaltered in the experimental flies (Fig. 5c). Genome sequencing revealed two polymorphisms in the RNAi target region (NCBI Acc. # KF192848-KF192876), potentially explaining the lack of a knock-down (see discussion).

### Insertion line dFoxP^3955^ is impaired in habit formation

Crystallization of birdsong shares a number of aspects with habit formation, a process induced by extended operant conditioning [55]. In humans, the mutations in the FOXP2 gene that lead to the developmental dyspraxia speech phenotype cause morphological alterations in cortico-striatal circuits [12], [17]. These circuits are also involved in habit formation [55], [56] and express FoxP2 in songbirds [57]. In flies, extended operant world-learning also produces habit formation: removing the color filters after sixteen minutes of training (but not after eight minutes) reveals a preference for the previously unpunished yaw torque domain [46]. Using flies with the *FoxP^3955^* insertion, we tested the hypothesis that the FoxP-dependent process of self-learning also underlies habit formation, but is inhibited during non-extended world-learning. Consistent with our hypothesis, *FoxP^3955^* mutants showed a significant impairment in habit formation after extended world-learning (Fig. 5d). Interestingly, the *FoxP^3955^* mutant flies do show a significant performance index after extended operant world-learning.

### Subtle morphological alterations in the brains of FoxP^3955^ mutant flies

FoxP mutations cause neuroanatomical alterations in multiple species [12], [16], [17], [57]–[59]. In flies, the general brain anatomy, structure, shape and relative position of the major neuropils did not differ markedly between wildtype and *FoxP^3955^* mutant flies (Fig. 6a). However, quantitative volumetric analysis of eleven major neuropils revealed that the most conspicuous of the recently identified ‘optic glomeruli’ [60] were significantly smaller in the mutant flies compared to Canton S controls (Fig. 6b). Moreover, a Principal Components Analysis (PCA) performed on the relative volume data of all neuropils also distinguished the *FoxP^3955^* mutant and control groups, in spite of spatial overlap of some individuals (Fig. 6c; for PCA results with data from optic glomeruli excluded, see Fig. S4). These results indicate that in flies, as in vertebrates, experimental manipulations of FoxP expression can lead to alterations in brain morphology.

## Discussion

### Genetic manipulation of the dFoxP locus

We selected three insertions within the *dFoxP* locus. Prior to testing, all three isoforms had been introgressed into the Canton S wild type strain, homogenizing the genetic background of all four lines. Two of the lines (*FoxP^f03746^* and *FoxP^c03619^*) did not express any detectable level of the novel intron-retention isoform mRNA. These two lines also showed a significant impairment in flight initiation and maintenance, suggesting that this isoform may be involved in flight performance (Fig. 2). A recent study supports the notion of a broader function of the different *dFoxP* isoforms in motor control [61] above and beyond operant self-learning. Coincidentally, this study and ours also serve to suggest that the *dFoxP*-dependent phenotype described in another, yet more recent report [62] may potentially also be due to motor issues, in contrast to the assertions of those authors. The operant self-learning phenotype of *FoxP^3955^* mutant flies (Fig. 3) was uncovered in the heterozygous state over a deficiency spanning the *dFoxP* locus, solidly tying the phenotype to this *dFoxP* manipulation (Fig. 4).

Line *FoxP^3955^* expressed a mutated isoform B mRNA, such that the putative isoform B protein was truncated and the C-terminal amino acid altered, potentially affecting protein function. There did not appear to be any change in the regulation of isoform B expression in this line as the expression levels were similar to the CS control strain on the mRNA level. This structural modification with its specific effect on operant self- but not world-learning is a reminder that many specific behavioral mutants are often due to structural mutations, commonly affecting only a subset of a gene's isoforms [63]. Targeting isoform B with a specific RNAi construct in order to probe for regulatory effects of *dFoxP*, yielded a phenocopy of the *FoxP^3955^* mutant behavioral phenotype, albeit without any detectable knock-down of the mRNA (Fig. 5). We cannot rule out that the knock-down did take place although at undetectable levels due to the already low levels of isoform B expression. The alternative also remains that hypothetical extra-neuronal expression could have masked any knock-down in the neuronal tissue. Finally, mismatches between the RNAi construct sequence and the target sequence can bias the RNAi process towards posttranscriptional silencing rather than mRNA degradation [64]–[67]. Sequencing of the strains used for the RNAi experiments indeed revealed two such polymorphisms leading to mismatches of the target region with the RNAi construct (see Results), potentially biasing the RNAi process towards sequestration.

It is thus plausible that the RNAi method employed here affected the protein, but not the overall mRNA levels of isoform B, explaining the behavioral phenotype. In the review process of this manuscript, an antibody against *dFoxP* isoform B was described [61], such that it will now be possible to test this hypothesis. Interestingly, none of the six currently available RNAi lines targeting the *dFoxP* locus show any detectable knock-down of any of the three isoforms (replicated in two different laboratories, each using a different pan-neuronal driver line, manuscript in prep.), despite one of them also showing a behavioral phenotype in two studies [61], [62], as well as mRNA knock-down using semi-quantitative PCR in the Lawton *et al.* work [61]. As we have observed similar *dFoxP* mRNA knock-downs using semi-quantitative PCR which were not confirmed by qPCR, we would cautiously speculate that the knock-down observed by Lawton *et al*. may be a false positive as well, with the behavioral phenotypes in both reports potentially explained by polymorphic mismatches in the target region. For these reasons, we can only tentatively conclude that besides the structural manipulations, also regulatory manipulations of only isoform B expression may affect operant self-learning. The most parsimonious explanation of the common phenotypes after various *dFoxP* manipulations is that *dFoxP* is indeed necessary for operant self-learning.

### Intact dFoxP gene required for normal brain development

Our volumetric analyses suggest a role of *Foxp^3955^* on brain development in *Drosophila,* analogous to the role FOXP2 plays in vertebrates [3], [4], [57], [68], [69]. Specifically, flies with the mutant *FoxP^3955^* allele have smaller optic glomeruli (Fig. 6). So far, very little is known about the functional role of optic glomeruli and, thus, of their relevance for operant self-learning. It has been hypothesized that optic glomeruli may form information hubs by virtue of containing the terminals of many projection neurons within the ipsilateral brain hemisphere [51]. Optic glomeruli have been discovered in dipterans and it is not known if other insect orders possess optic glomeruli. Nevertheless, it is interesting to note that a conspicuous cluster of *AmFoxP*-expressing neurons is located near the optic lobes of the honeybee [70]. Notably, no significant change was observed in the protocerebral bridge, a neuropil in which a recently developed *dFoxP*-GAL4 driver line was reported to express [61].

It deserves to be emphasized, however, that the overall structure of the brains of *FoxP^3955^* mutant flies appears completely normal to the human eye. Only numerical analyses are capable of detecting the subtle changes this mutation causes to brain anatomy. More severe alleles of the *dFoxP* locus have been observed to lead to more severe anatomical defects (Troy Zars, unpublished observation).

### FoxP is specifically involved in self-learning

We targeted *dFoxP* with our genetic manipulations because of the conserved role of the FoxP gene family in vocal learning. We then tested these manipulations each in two very similar operant learning paradigms that differ in their conceptual similarity to language acquisition.

Operant self-learning in *Drosophila* parallels the operant feedback structure of vocal learning in humans or songbirds in that no other external cues are contingent upon the feedback (Fig. 1). This conceptual similarity is now supported by the parallel biological similarity of both vocal and operant self-learning requiring FoxP function (e.g., [11]–[13], [23], [71] and Figs. 3, 4, 5). Interestingly, Protein Kinase C (PKC), the only other known molecular component of the self-learning mechanism [32], [72], [73], has also been implicated in vocal learning in songbirds [74], [75].

The technically all but identical operant world-learning task, in contrast, not only differs conceptually from vocal learning – the behavior is also controlling an external cue (the colors) – but the biological requirements are also different: neither *dFoxP* (Figs. 3, 5) nor PKC [32] function is necessary. Recent experiments reporting unaffected Pavlovian conditioning in *FoxP^3955^* mutant flies [62] confirm the notion that *FoxP* is specifically required for self-learning. PKC is not required for other forms of world-learning in flies either [76], [77], but in other model systems, the data are less straightforward, with varying requirements of varying PKC isoforms for various phases of memory induction and/or maintenance having been reported, depending on preparation, type of training, time of testing, brain region and method of PKC manipulation. To our knowledge, there is only one experiment where PKC requirement has been compared between self- and world-learning. This experiment in mice supports the view that PKC-activity in the cerebellum is not required for world-learning but for self-learning [72], analogous to the requirements in flies [32]. Conversely, the components that are required for operant world-learning in flies are the same that are required for many other forms of learning in many other model systems, such as classical (Pavlovian) conditioning, sensitization or some forms of habituation, e.g., the type 1 adenylyl cyclase [78]–[90], encoded by the fly gene *rutabaga*, which is dispensable for self-learning [32], [73].

Thus, we postulate that the converging evidence from multiple model systems concerning PKC and *FoxP* provides first insights into a core set of mechanisms that are specifically required for operant self-learning, and not for other forms of (associative) learning, such as world learning.

### Self-learning and habit formation share the same biochemical substrate

We found that not only self-learning but also habit formation is impaired in *FoxP^3955^* mutant flies (Fig. 5), indicating that habit formation may be mediated by the same biochemical processes as operant self-learning, as had been hypothesized before [46], [47]. If wild-type flies are trained in world-learning for eight minutes (i.e., with colors) and then tested for their turning preference in the absence of the colors, there is no preference, demonstrating a hierarchical learning system where external cues are preferentially memorized/retrieved (world-learning) over behavioral cues (self-learning)[46]–[48]. However, if training in the world-learning situation (i.e., with colors) is extended to 16 minutes and then the same preference test for turning is performed (i.e., without the colors), then a preference for turning towards the previously unpunished direction can be observed [46]. Because self-learning is manifest already after eight minutes of training (i.e., without colors), the presence and learning of the colors during training must have inhibited self-learning, apparently via a neuropil in the fly brain termed mushroom-bodies [46]. Extended training can overcome this inhibition and lead to habit formation. These habits lead to reduced generalization in ‘habit interference’ experiments when an orthogonal behavior is used to control the colors after extended world-learning [46]. Here, we show that flies with a mutated *dFoxP* gene are impaired in habit formation (Fig. 5), i.e., extended operant world-learning leads to a reduced preference for the previously unpunished yaw torque domain in a test without colors. Interestingly, the preference for the unpunished turning direction in the mutant flies is statistically significant, suggesting that perhaps flies with the mutated gene product are still able to encode some memory, albeit at greatly reduced efficiency. This finding also indicates that perhaps extended self-learning (i.e., without colors) might also be able to yield significant learning scores in *FoxP^3955^* mutant flies. We will therefore test these flies not only in extended self-learning, but also in habit interference experiments in future work.

Vocal learning has been characterized as an automatization of behavior, akin to habit formation in non-vocal mammals [55]. Analogous to the conceptual and biological similarity of self-learning across taxa, both vocal learning and habit formation in vertebrates also share biological substrates in cortico-striatal circuits, where FoxP2 is expressed [12], [17], [55]–[57]. Our finding that the *FoxP^3955^* allele is also involved in habit formation in invertebrates not only further supports the homology between PKC/FoxP-mediated operant self-learning in flies and vocal learning in birds and humans, but also prompts the hypothesis that habit formation may require a FoxP gene in vertebrates as well.

### Self-learning as an exaptation for language acquisition

Thus, we can draw from several bodies of evidence spanning multiple vertebrate and invertebrate model systems and humans, when we conclude that our results strongly support the hypothesis that the FoxP-dependent component of language evolved from an ancestral operant self-learning mechanism. Interestingly, preliminary data from non-vocal vertebrate motor learning further corroborate this hypothesis [91], [92]. Moreover, following this extension of the ‘motor learning hypothesis’ of FoxP function [23], [24], one may predict language and/or motor skill deficits in patients with mutations in other FOXP paralogues, anticipating that some of the ancestral function is conserved. Indeed, the symptoms of patients with mutations in the closest paralogue of FOXP2, FOXP1, include language and motor skill impairments [18], [19], [21], [22]. In fact, the *Drosophila* isoform probably involved in self-learning is not present in FoxP2 genes, but in FoxP1 [36]. Because FoxP gene products form homo- as well as heterodimers [93], [94], it is tempting to speculate that all three brain-expressed FoxP paralogues (1, 2 and 4 [95]) may perform similar tasks in a degenerate fashion, with the neuronal circuits controlling language being more susceptible to disruption than other FoxP-expressing circuits.

Autism spectrum disorders and schizophrenia are being discussed as diametric malfunctions on a continuous scale of ‘sense of self’ [5], [96]. Given the implication of members of the FoxP gene family in both disorders [5]–[10], [18], [22], [97], [98], it is interesting to note that the operant self-learning mechanism appears to be selectively engaged when the content of the learning task concerns the organism itself and not when it concerns non-self entities (Figs. 3, 5).

In contrast to Chomsky's assertion that it is “completely meaningless to speak of extrapolating this concept of operant to ordinary verbal behavior” [99], we were able to show that a transcription factor known for its specific involvement in language is also involved in operant conditioning, specifically operant self-learning. This result is consistent with Skinner's description of language as an operant behavior [27], [100], while nevertheless espousing Chomsky's view of inborn components in language acquisition [99]. In particular, our results emphasize the speech component in the evolution of language: “In many respects the story of the evolution of language must begin with the evolution of serial motor activity and its nervous control” [101].

## Supporting Information

Figure S1 Four aspects of flight behaviour in wild type and mutant flies. For panels A-C the black squares indicate the median, the boxes signify the 25 and the 75 percentiles, and the error bars range from the 15 to the 85 percentiles. A – Flight duration until the first stop for wild type (Canton S, yellow box), *FoxP^3955^* (orange box) and the other two insertion lines (grey boxes). B – Average duration of flight bouts for wild type and mutant lines. C – Total flight duration as in Fig. 1. D – Mean number of stimuli to which the flies responded with flight bouts before they did not respond to 3 consecutive stimuli (errors are s.e.m.s). Numbers in bars – number of animals. Asterisks – significant (p<0.05) differences to wild type Canton S in post-hoc tests after a significant Kruskal-Wallis ANOVA.
(EPS)

Figure S2 Separating the genetic control groups still shows the RNAi effect in operant self-learning. Both genetic control lines show significant learning scores of 0.37 and above (t-tests against zero, elav/+: t = 2.26, p = 0.038; RNAi/+: t = 3.53, p = 0.004). In contrast, the flies from the strain in which the elav driver expressed the isoform B-specific RNAi construct in all neurons, fails to reach even half of the lowest control score with a PI of less than 0.17, which cannot be distinguished from a PI of zero (t-test against zero; t = 0.141, P = 0.166).
(EPS)

Figure S3 Subtle morphological alterations in the brains of FoxP^3955^ mutants. **a**, Three-dimensional surface renderings of typical fly brains from wild type Canton S (**a1**) and *FoxP^3955^* mutants (**a2**). In the online version, clicking on the reconstructions will activate the 3D features of the figure and allow for interactions with the object in space. The different neuropil areas can be selected in the pop-up menu. **b**, Quantitative volumetric analysis of eleven major neuropils (M – medulla, L – lobula, LP – lobula plate, MB – mushroom bodies, AL – antennal lobes, FB – fan-shaped body, OT – optic tubercle, EB – ellipsoid body, OG – optic glomeruli (purple in **a**), PB – protocerebral bridge, N – noduli) revealed a significant reduction in the volume of the optic glomeruli in *FoxP^3955^* flies (Mann-Whitney U-Test, U = 2.0, p<0.002). The volume of the remaining neuropils (denoted PL – protocerebral lobes) did not differ significantly. Asterisk – significant difference with a Bonferroni-corrected level of p<0.004. Black stripes – median, boxes – 25–75% percentiles, whiskers – total range. Grey boxes indicate *FoxP^3955^*, white boxes Canton S. **c**, Principal Components Analysis of the volumetric data. Plotted are the factor loadings of the individual flies on the two first components. Colored bars indicate means and standard errors (PC). Factor loadings are significantly different between Canton S and *Foxp^3955^* for PC1 (Mann-Whitney U-Test, U = 52.0, p<0.04), but fail to reach significance for PC2. Number of brains analyzed: 7 (Canton S) and 9 (*Foxp^3955^*).
(PDF)

Figure S4 PCA on relative volume data with optic glomeruli excluded from the analysis. The general distribution of the individual fly brains remains roughly similar to the PCA on all data (Fig. 6), suggesting that the exclusion of the volume data from the optic glomeruli does not drastically alter the general picture of anatomical differences between the mutant and wild type flies also outside of the optic glomeruli. However, the ANOVA on the differences between the two groups now fails to reach significance, emphasizing the importance of the volume difference we found in the optic glomeruli.
(EPS)

## References

[1] Lai CS, Fisher SE, Hurst JA, Vargha-Khadem F, Monaco AP (2001) A forkhead-domain gene is mutated in a severe speech and language disorder. Nature 413: 519–523 10.1038/35097076 11586359

[2] Enard W, Przeworski M, Fisher SE, Lai CSL, Wiebe V, et al (2002) Molecular evolution of FOXP2, a gene involved in speech and language. Nature 418: 869–872 10.1038/nature01025 12192408

[3] Konopka G, Bomar JM, Winden K, Coppola G, Jonsson ZO, et al (2009) Human-specific transcriptional regulation of CNS development genes by FOXP2. Nature 462: 213–217 10.1038/nature08549 19907493 PMC2778075

[4] Spaniel F, Horáček J, Tintěra J, Ibrahim I, Novák T, et al (2011) Genetic variation in FOXP2 alters grey matter concentrations in schizophrenia patients. Neurosci Lett 493: 131–135 10.1016/j.neulet.2011.02.024 21334420

[5] Crespi B, Stead P, Elliot M (2010) Evolution in health and medicine Sackler colloquium: Comparative genomics of autism and schizophrenia. Proc Natl Acad Sci U S A 107 Suppl: 1736–1741 10.1073/pnas.0906080106 PMC2868282 19955444

[6] Li H, Yamagata T, Mori M, Momoi MY (2005) Absence of causative mutations and presence of autism-related allele in FOXP2 in Japanese autistic patients. Brain Dev 27: 207–210 10.1016/j.braindev.2004.06.002 15737702

[7] Gong X, Jia M, Ruan Y, Shuang M, Liu J, et al (2004) Association between the FOXP2 gene and autistic disorder in Chinese population. Am J Med Genet B Neuropsychiatr Genet 127B: 113–116 10.1002/ajmg.b.20162 15108192

[8] Li T, Zeng Z, Zhao Q, Wang T, Huang K, et al (2013) FoxP2 is significantly associated with schizophrenia and major depression in the Chinese Han population. World J Biol Psychiatry 14: 146–150 10.3109/15622975.2011.615860 22404659

[9] McCarthy-Jones S, Green MJ, Scott RJ, Tooney PA, Cairns MJ, et al.. (2013) Preliminary evidence of an interaction between the FOXP2 gene and childhood emotional abuse predicting likelihood of auditory verbal hallucinations in schizophrenia. J Psychiatr Res. doi:10.1016/j.jpsychires.2013.11.012. 10.1016/j.jpsychires.2013.11.012 24360035

[10] Toma C, Hervás A, Torrico B, Balmaña N, Salgado M, et al (2013) Analysis of two language-related genes in autism: a case-control association study of FOXP2 and CNTNAP2. Psychiatr Genet 23: 82–85 10.1097/YPG.0b013e32835d6fc6 23277129

[11] Fisher SE, Scharff C (2009) FOXP2 as a molecular window into speech and language. Trends Genet 25: 166–177 10.1016/j.tig.2009.03.002 19304338

[12] Enard W (2011) FOXP2 and the role of cortico-basal ganglia circuits in speech and language evolution. Curr Opin Neurobiol 21: 415–424 10.1016/j.conb.2011.04.008 21592779

[13] Haesler S, Rochefort C, Georgi B, Licznerski P, Osten P, et al (2007) Incomplete and Inaccurate Vocal Imitation after Knockdown of FoxP2 in Songbird Basal Ganglia Nucleus Area X. PLoS Biol 5: 12 10.1371/journal.pbio.0050321 PMC2100148 18052609

[14] Berwick RC, Friederici AD, Chomsky N, Bolhuis JJ (2013) Evolution, brain, and the nature of language. Trends Cogn Sci 17: 89–98 10.1016/j.tics.2012.12.002 23313359

[15] Bacon C, Rappold GA (2012) The distinct and overlapping phenotypic spectra of FOXP1 and FOXP2 in cognitive disorders. Hum Genet. doi:10.1007/s00439-012-1193-z. 10.1007/s00439-012-1193-z PMC3470686 22736078

[16] Groszer M, Keays DA, Deacon RMJ, de Bono JP, Prasad-Mulcare S, et al (2008) Impaired Synaptic Plasticity and Motor Learning in Mice with a Point Mutation Implicated in Human Speech Deficits. Curr Biol 18: 354–362. 18328704 10.1016/j.cub.2008.01.060 PMC2917768

[17] Scharff C, Petri J (2011) Evo-devo, deep homology and FoxP2: implications for the evolution of speech and language. Philos Trans R Soc Lond B Biol Sci 366: 2124–2140 10.1098/rstb.2011.0001 21690130 PMC3130369

[18] Hamdan FF, Daoud H, Rochefort D, Piton A, Gauthier J, et al (2010) De novo mutations in FOXP1 in cases with intellectual disability, autism, and language impairment. Am J Hum Genet 87: 671–678 10.1016/j.ajhg.2010.09.017 20950788 PMC2978954

[19] Newbury DF, Monaco AP (2010) Genetic advances in the study of speech and language disorders. Neuron 68: 309–320 10.1016/j.neuron.2010.10.001 20955937 PMC2977079

[20] Kurt S, Fisher SE, Ehret G (2012) Foxp2 mutations impair auditory-motor association learning. PLoS One 7: e33130 10.1371/journal.pone.0033130 22412993 PMC3296769

[21] Horn D (2012) Mild to Moderate Intellectual Disability and Significant Speech and Language Deficits in Patients with FOXP1 Deletions and Mutations. Mol Syndromol 2: 213–216 10.1159/000330916 22670142 PMC3366708

[22] Palumbo O, D′Agruma L, Minenna AF, Palumbo P, Stallone R, et al (2013) 3p14.1 de novo microdeletion involving the FOXP1 gene in an adult patient with autism, severe speech delay and deficit of motor coordination. Gene 516: 107–113 10.1016/j.gene.2012.12.073 23287644

[23] Bolhuis JJ, Okanoya K, Scharff C (2010) Twitter evolution: converging mechanisms in birdsong and human speech. Nat Rev Neurosci 11: 747–759 10.1038/nrn2931 20959859

[24] Feenders G, Liedvogel M, Rivas M, Zapka M, Horita H, et al (2008) Molecular mapping of movement-associated areas in the avian brain: a motor theory for vocal learning origin. PLoS One 3: e1768 10.1371/journal.pone.0001768 18335043 PMC2258151

[25] Corballis MC (2009) The evolution of language. Ann N Y Acad Sci 1156: 19–43 10.1111/j.1749-6632.2009.04423.x 19338501

[26] Teramitsu I, White SA (2008) Motor Learning: The FoxP2 Puzzle Piece. Curr Biol 18: 0. 10.1016/j.cub.2008.02.048 PMC2699589 18430631

[27] Naour P (2009) E.O. Wilson and B.F. Skinner: A Dialogue Between Sociobiology and Radical Behaviorism. New York, NY: Springer New York. doi:10.1007/978-0-387-89462-1.

[28] Marler P (1991) Song-learning behavior: The interface with neuroethology. Trends Neurosci 14: 199–206 10.1016/0166-2236(91)90106-5 1713722

[29] Fee MS (2014) The role of efference copy in striatal learning. Curr Opin Neurobiol 25C: 194–200 10.1016/j.conb.2014.01.012 PMC4153469 24566242

[30] Mooney R (2004) Synaptic mechanisms for auditory-vocal integration and the correction of vocal errors. Ann N Y Acad Sci 1016: 476–494 10.1196/annals.1298.011 15313791

[31] Wolf R, Heisenberg M (1991) Basic organization of operant behavior as revealed in Drosophila flight orientation. J Comp Physiol A 169: 699–705 10.1007/BF00194898 1795235

[32] Brembs B, Plendl W (2008) Double dissociation of pkc and ac manipulations on operant and classical learning in drosophila. Curr Biol 18: 1168–1171 10.1016/j.cub.2008.07.041 18674907

[33] Guo A, Liu L, Xia S-Z, Feng C-H, Wolf R, et al (1996) Conditioned visual flight orientation in Drosophila; Dependence on age, practice and diet. Learn Mem 3: 49–59. 10456076 10.1101/lm.3.1.49

[34] Brembs B (2008) Operant learning of drosophila at the torque meter. J Vis Exp: 2006–2008. doi:10.3791/731. 10.3791/731 PMC2956216 19066552

[35] Brand AH, Perrimon N (1993) Targeted gene expression as a means of altering cell fates and generating dominant phenotypes. 118: 401–415. 10.1242/dev.118.2.401 8223268

[36] Santos ME, Athanasiadis A, Leitão AB, DuPasquier L, Sucena E (2011) Alternative splicing and gene duplication in the evolution of the FoxP gene subfamily. Mol Biol Evol 28: 237–247 10.1093/molbev/msq182 20651048 PMC3002244

[37] Ponton F, Chapuis M-P, Pernice M, Sword GA, Simpson SJ (2011) Evaluation of potential reference genes for reverse transcription-qPCR studies of physiological responses in Drosophila melanogaster. J Insect Physiol 57: 840–850 10.1016/j.jinsphys.2011.03.014 21435341

[38] Higgins D, Sharp P (1988) CLUSTAL: a package for performing multiple sequence alignment on a microcomputer. Gene 73: 237–244 10.1016/0378-1119(88)90330-7 3243435

[39] Letunic I, Bork P (2007) Interactive Tree Of Life (iTOL): an online tool for phylogenetic tree display and annotation. Bioinformatics 23: 127–128 10.1093/bioinformatics/btl529 17050570

[40] Brembs B, Christiansen F, Pflüger HJ, Duch C (2007) Flight initiation and maintenance deficits in flies with genetically altered biogenic amine levels. J Neurosci 27: 11122–11131 10.1523/JNEUROSCI.2704-07.2007 17928454 PMC6672854

[41] Götz KG (1964) Optomotorische Untersuchung des visuellen systems einiger Augenmutanten der Fruchtfliege Drosophila. Kybernetik 2: 77–92 10.1007/BF00288561 5833196

[42] Brembs B, Hempel De Ibarra N (2006) Different parameters support generalization and discrimination learning in drosophila at the flight simulator. Learn Mem 13: 629–637 10.1101/lm.319406 17015859 PMC1783617

[43] Tang S, Juusola M (2010) Intrinsic Activity in the Fly Brain Gates Visual Information during Behavioral Choices. PLoS One 5: e14455 10.1371/journal.pone.0014455 21209935 PMC3012687

[44] Maguire LP, Szilagyi S, Scholten RE (2004) High performance laser shutter using a hard disk drive voice-Coil actuator. Rev Sci Instrum 75: 3077. 10.1063/1.2437199 17578150

[45] Brembs B, Heisenberg M (2000) The Operant and the Classical in Conditioned Orientation of Drosophila melanogaster at the Flight Simulator. Learn Mem 7: 104–115 10.1101/lm.7.2.104 10753977 PMC311324

[46] Brembs B (2009) Mushroom bodies regulate habit formation in Drosophila. Curr Biol 19: 1351–1355 10.1016/j.cub.2009.06.014 19576773

[47] Colomb J, Brembs B (2010) The biology of psychology: Simple conditioning? Commun Integr Biol 3: 142–145 10.4161/cib.3.2.10334 20585506 PMC2889970

[48] Brembs B (2011) Spontaneous decisions and operant conditioning in fruit flies. Behav Processes 87: 157–164 10.1016/j.beproc.2011.02.005 21392558

[49] Seki Y, Rybak J, Wicher D, Sachse S, Hansson BS (2010) Physiological and morphological characterization of local interneurons in the Drosophila antennal lobe. J Neurophysiol 104: 1007–1019 10.1152/jn.00249.2010 20505124

[50] Wagh DA, Rasse TM, Asan E, Hofbauer A, Schwenkert I, et al (2006) Bruchpilot, a protein with homology to ELKS/CAST, is required for structural integrity and function of synaptic active zones in Drosophila. Neuron 49: 833–844 10.1016/j.neuron.2006.02.008 16543132

[51] Chiang A-S, Lin C-Y, Chuang C-C, Chang H-M, Hsieh C-H, et al (2011) Three-dimensional reconstruction of brain-wide wiring networks in Drosophila at single-cell resolution. Curr Biol 21: 1–11 10.1016/j.cub.2010.11.056 21129968

[52] Rybak J, Kuβ A, Lamecker H, Zachow S, Hege H-C, et al.. (2010) The Digital Bee Brain: Integrating and Managing Neurons in a Common 3D Reference System. Front Syst Neurosci 4. doi:10.3389/fnsys.2010.00030. 10.3389/fnsys.2010.00030 PMC2935790 20827403

[53] Gabut M, Samavarchi-Tehrani P, Wang X, Slobodeniuc V, O′Hanlon D, et al (2011) An alternative splicing switch regulates embryonic stem cell pluripotency and reprogramming. Cell 147: 132–146 10.1016/j.cell.2011.08.023 21924763

[54] Maye A, Hsieh C, Sugihara G, Brembs B (2007) Order in spontaneous behavior. PLoS One 2: e443 10.1371/journal.pone.0000443 17505542 PMC1865389

[55] Costa RM (2011) A selectionist account of de novo action learning. Curr Opin Neurobiol 21: 579–586 10.1016/j.conb.2011.05.004 21641793

[56] Balleine BW, O′Doherty JP (2010) Human and rodent homologies in action control: corticostriatal determinants of goal-directed and habitual action. Neuropsychopharmacology 35: 48–69 10.1038/npp.2009.131 19776734 PMC3055420

[57] Schulz SB, Haesler S, Scharff C, Rochefort C (2010) Knockdown of FoxP2 alters spine density in Area X of the zebra finch. Genes Brain Behav 9: 732–740 10.1111/j.1601-183X.2010.00607.x 20528955

[58] Enard W, Gehre S, Hammerschmidt K, Hölter SM, Blass T, et al (2009) A humanized version of Foxp2 affects cortico-basal ganglia circuits in mice. Cell 137: 961–971 10.1016/j.cell.2009.03.041 19490899

[59] Reimers-Kipping S, Hevers W, Pääbo S, Enard W (2011) Humanized Foxp2 specifically affects cortico-basal ganglia circuits. Neuroscience 175: 75–84 10.1016/j.neuroscience.2010.11.042 21111790

[60] Strausfeld NJ, Okamura J-Y (2007) Visual system of calliphorid flies: organization of optic glomeruli and their lobula complex efferents. J Comp Neurol 500: 166–188 10.1002/cne.21196 17099891

[61] Lawton KJ, Wassmer TL, Deitcher DL (2014) Conserved role of Drosophila melanogaster FoxP in motor coordination and courtship song. Behav Brain Res 268: 213–221 10.1016/j.bbr.2014.04.009 24747661

[62] DasGupta S, Ferreira CH, Miesenbock G (2014) FoxP influences the speed and accuracy of a perceptual decision in Drosophila. Science (80-) 344: 901–904 10.1126/science.1252114 PMC4206523 24855268

[63] Pflugfelder GO (1998) Genetic lesions in Drosophila behavioural mutants. Behav Brain Res 95: 3–15. 9754871 10.1016/s0166-4328(97)00204-0

[64] Chu C, Rana TM (2006) Translation repression in human cells by microRNA-induced gene silencing requires RCK/p54. PLoS Biol 4: e210 10.1371/journal.pbio.0040210 16756390 PMC1475773

[65] Carthew RW, Sontheimer EJ (2009) Origins and Mechanisms of miRNAs and siRNAs. Cell 136: 642–655 10.1016/j.cell.2009.01.035 19239886 PMC2675692

[66] Tomari Y, Zamore PD (2005) Perspective: machines for RNAi. Genes Dev 19: 517–529 10.1101/gad.1284105 15741316

[67] Fukaya T, Tomari Y (2012) MicroRNAs mediate gene silencing via multiple different pathways in drosophila. Mol Cell 48: 825–836 10.1016/j.molcel.2012.09.024 23123195

[68] Spiteri E, Konopka G, Coppola G, Bomar J, Oldham M, et al (2007) Identification of the transcriptional targets of FOXP2, a gene linked to speech and language, in developing human brain. Am J Hum Genet 81: 1144–1157 10.1086/522237 17999357 PMC2276350

[69] Vernes SC, Oliver PL, Spiteri E, Lockstone HE, Puliyadi R, et al (2011) Foxp2 regulates gene networks implicated in neurite outgrowth in the developing brain. PLoS Genet 7: e1002145 10.1371/journal.pgen.1002145 21765815 PMC3131290

[70] Kiya T, Itoh Y, Kubo T (2008) Expression analysis of the FoxP homologue in the brain of the honeybee, Apis mellifera. Insect Mol Biol 17: 53–60 10.1111/j.13652583.2008.00775.x 18237284

[71] Lai CSL, Fisher SE, Hurst JA, Vargha-khadem F, Monaco AP (2001) A forkhead-domain gene is mutated in a severe speech and language disorder. Nature 413: 519–523. 11586359 10.1038/35097076

[72] Rochefort C, Arabo A, Andre M, Poucet B, Save E, et al (2011) Cerebellum Shapes Hippocampal Spatial Code. Science (80-) 334: 385–389 10.1126/science.1207403 22021859

[73] Lorenzetti FD, Baxter DA, Byrne JH (2008) Molecular Mechanisms Underlying a Cellular Analog of Operant Reward Learning. Neuron 59: 815–828. 18786364 10.1016/j.neuron.2008.07.019 PMC2603610

[74] Sakaguchi H, Yamaguchi A (1997) Early song-deprivation affects the expression of protein kinase C in the song control nuclei of the zebra finch during a sensitive period of song learning. Neuroreport 8: 2645–2650. 9295093 10.1097/00001756-199708180-00002

[75] Yoshida Y, Yamada T, Sakaguchi H (2003) Activation of protein kinase C by the error signal from a basal ganglia-forebrain circuit in the zebra finch song control nuclei. Neuroreport 14: 645–649 10.1097/01.wnr.0000059995.72968.e0 12657904

[76] Kane NS, Robichon A, Dickinson JA, Greenspan RJ (1997) Learning without performance in PKC-deficient Drosophila. Neuron 18: 307–314. 9052800 10.1016/s0896-6273(00)80270-6

[77] Drier EA, Tello MK, Cowan M, Wu P, Blace N, et al (2002) Memory enhancement and formation by atypical PKM activity in Drosophila melanogaster. Nat Neurosci 5: 316–324. 11914720 10.1038/nn820

[78] Duerr JS, Quinn WG (1982) Three Drosophila Mutations That Block Associative Learning Also Affect Habituation and Sensitization. Proc Natl Acad Sci U S A 79: 3646–3650. 6808513 10.1073/pnas.79.11.3646 PMC346480

[79] Engel JE, Wu CF (1996) Altered habituation of an identified escape circuit in Drosophila memory mutants. J Neurosci 16: 3486–3499. 8627381 10.1523/JNEUROSCI.16-10-03486.1996 PMC6579151

[80] Zars T, Wolf R, Davis R, Heisenberg M (2000) Tissue-specific expression of a type I adenylyl cyclase rescues the rutabaga mutant memory defect: in search of the engram. Learn Mem 7: 18–31. 10706599 10.1101/lm.7.1.18 PMC311318

[81] Liu G, Seiler H, Wen A, Zars T, Ito K, et al (2006) Distinct memory traces for two visual features in the Drosophila brain. Nature 439: 551–556. 16452971 10.1038/nature04381

[82] Zars T, Fischer M, Schulz R, Heisenberg M (2000) Localization of a short-term memory in Drosophila. Science (80-) 288: 672–675. 10.1126/science.288.5466.672 10784450

[83] Gervasi N, Tchénio P, Preat T (2010) PKA dynamics in a Drosophila learning center: coincidence detection by rutabaga adenylyl cyclase and spatial regulation by dunce phosphodiesterase. Neuron 65: 516–529 10.1016/j.neuron.2010.01.014 20188656

[84] Margulies C, Tully T, Dubnau J (2005) Deconstructing memory in Drosophila. Curr Biol 15: 0–13. 10.1016/j.cub.2005.08.024 PMC3044934 16139203

[85] Keene AC, Waddell S (2007) Drosophila olfactory memory: single genes to complex neural circuits. Nat Rev Neurosci 8: 341–354. 17453015 10.1038/nrn2098

[86] Abrams TW, Karl KA, Kandel ER (1991) Biochemical studies of stimulus convergence during classical conditioning in Aplysia: dual regulation of adenylate cyclase by Ca2+/calmodulin and transmitter. J Neurosci 11: 2655–2665. 1679120 10.1523/JNEUROSCI.11-09-02655.1991 PMC6575265

[87] Engel JE, Wu C-F (2009) Neurogenetic approaches to habituation and dishabituation in Drosophila. Neurobiol Learn Mem 92: 166–175 10.1016/j.nlm.2008.08.003 18765288 PMC2730516

[88] Wei F, Qiu CS, Kim SJ, Muglia L, Maas JW, et al (2002) Genetic elimination of behavioral sensitization in mice lacking calmodulin-stimulated adenylyl cyclases. Neuron 36: 713–726. 12441059 10.1016/s0896-6273(02)01019-x

[89] Wong ST, Athos J, Figueroa XA, Pineda VV, Schaefer ML, et al (1999) Calcium-stimulated adenylyl cyclase activity is critical for hippocampus-dependent long-term memory and late phase LTP. Neuron 23: 787–798. 10482244 10.1016/s0896-6273(01)80036-2

[90] Ueda A, Wu C-F (2012) Cyclic Adenosine Monophosphate Metabolism in Synaptic Growth, Strength, and Precision: Neural and Behavioral Phenotype-Specific Counterbalancing Effects between dnc Phosphodiesterase and rut Adenylyl Cyclase Mutations. J Neurogenet 26: 64–81 10.3109/01677063.2011.652752 22380612 PMC3572794

[91] Callaway E (2011) “Language gene” speeds learning. Nature. doi:10.1038/nature.2011.9395.

[92] French CA, Jin X, Campbell TG, Gerfen E, Groszer M, et al.. (2011) An aetiological Foxp2 mutation causes aberrant striatal activity and alters plasticity during skill learning. Mol Psychiatry. doi:10.1038/mp.2011.105. 10.1038/mp.2011.105 PMC3481071 21876543

[93] Li S, Weidenfeld J, Morrisey EE (2004) Transcriptional and DNA binding activity of the Foxp1/2/4 family is modulated by heterotypic and homotypic protein interactions. Mol Cell Biol 24: 809–822. 14701752 10.1128/MCB.24.2.809-822.2004 PMC343786

[94] Chae W-J, Henegariu O, Lee S-K, Bothwell ALM (2006) The mutant leucine-zipper domain impairs both dimerization and suppressive function of Foxp3 in T cells. Proc Natl Acad Sci U S A 103: 9631–9636 10.1073/pnas.0600225103 16769892 PMC1480458

[95] Takahashi H, Takahashi K, Liu F-C (2009) FOXP genes, neural development, speech and language disorders. Adv Exp Med Biol 665: 117–129. 20429420 10.1007/978-1-4419-1599-3_9

[96] Crespi B, Badcock C (2008) Psychosis and autism as diametrical disorders of the social brain. Behav Brain Sci 31: 241–61 discussion 261–320 10.1017/S0140525X08004214 18578904

[97] Bowers JM, Konopka G (2012) The role of the FOXP family of transcription factors in ASD. Dis Markers 33: 251–260 10.3233/DMA-2012-0919 22960337 PMC3810785

[98] Chien W-H, Gau SS-F, Chen C-H, Tsai W-C, Wu Y-Y, et al (2013) Increased gene expression of FOXP1 in patients with autism spectrum disorders. Mol Autism 4: 23 10.1186/2040-2392-4-23 23815876 PMC3723673

[99] Chomsky N (1959) A Review of B. F. Skinner's Verbal Behavior. Language (Baltim) 35: 26–58.

[100] Skinner BF (1957) Verbal Behavior. Copley Publishing Group

[101] Steklis HD, Harnad SR (1976) From hand to mouth: some critical stages in the evolution of language. Ann N Y Acad Sci 280: 445–455. 827953 10.1111/j.1749-6632.1976.tb25508.x

