# Supplementary material for: Drosophila FoxP Mutants Are Deficient in Operant Self-Learning
Source: PLoS One. 2014 Jun 25;9(6):e100648. doi: 10.1371/journal.pone.0100648 (PMC4070984; doi:10.1371/journal.pone.0100648)
Supplement: Figure S3 — Subtle morphological alterations in the brains of FoxP3955 mutants. a, Three-dimensional surface renderings of typical fly brains from wild type Canton S (a1) and FoxP3955 mutants (a2). In the online version, clicking on the reconstructions will activate the 3D features of the figure and allow for interactions with the object in space. The different neuropil areas can be selected in the pop-up menu. b, Quantitative volumetric analysis of eleven major neuropils (M – medulla, L – lobula, LP – lobula plate, MB – mushroom bodies, AL – antennal lobes, FB – fan-shaped body, OT – optic tubercle, EB – ellipsoid body, OG – optic glomeruli (purple in a), PB – protocerebral bridge, N – noduli) revealed a significant reduction in the volume of the optic glomeruli in FoxP3955 flies (Mann-Whitney U-Test, U = 2.0, p<0.002). The volume of the remaining neuropils (denoted PL – protocerebral lobes) did not differ significantly. Asterisk – significant difference with a Bonferroni-corrected level of p<0.004. Black stripes – median, boxes – 25–75% percentiles, whiskers – total range. Grey boxes indicate FoxP3955, white boxes Canton S. c, Principal Components Analysis of the volumetric data. Plotted are the factor loadings of the individual flies on the two first components. Colored bars indicate means and standard errors (PC). Factor loadings are significantly different between Canton S and Foxp3955 for PC1 (Mann-Whitney U-Test, U = 52.0, p<0.04), but fail to reach significance for PC2. Number of brains analyzed: 7 (Canton S) and 9 (Foxp3955). (PDF) [file pone.0100648.s003.pdf]

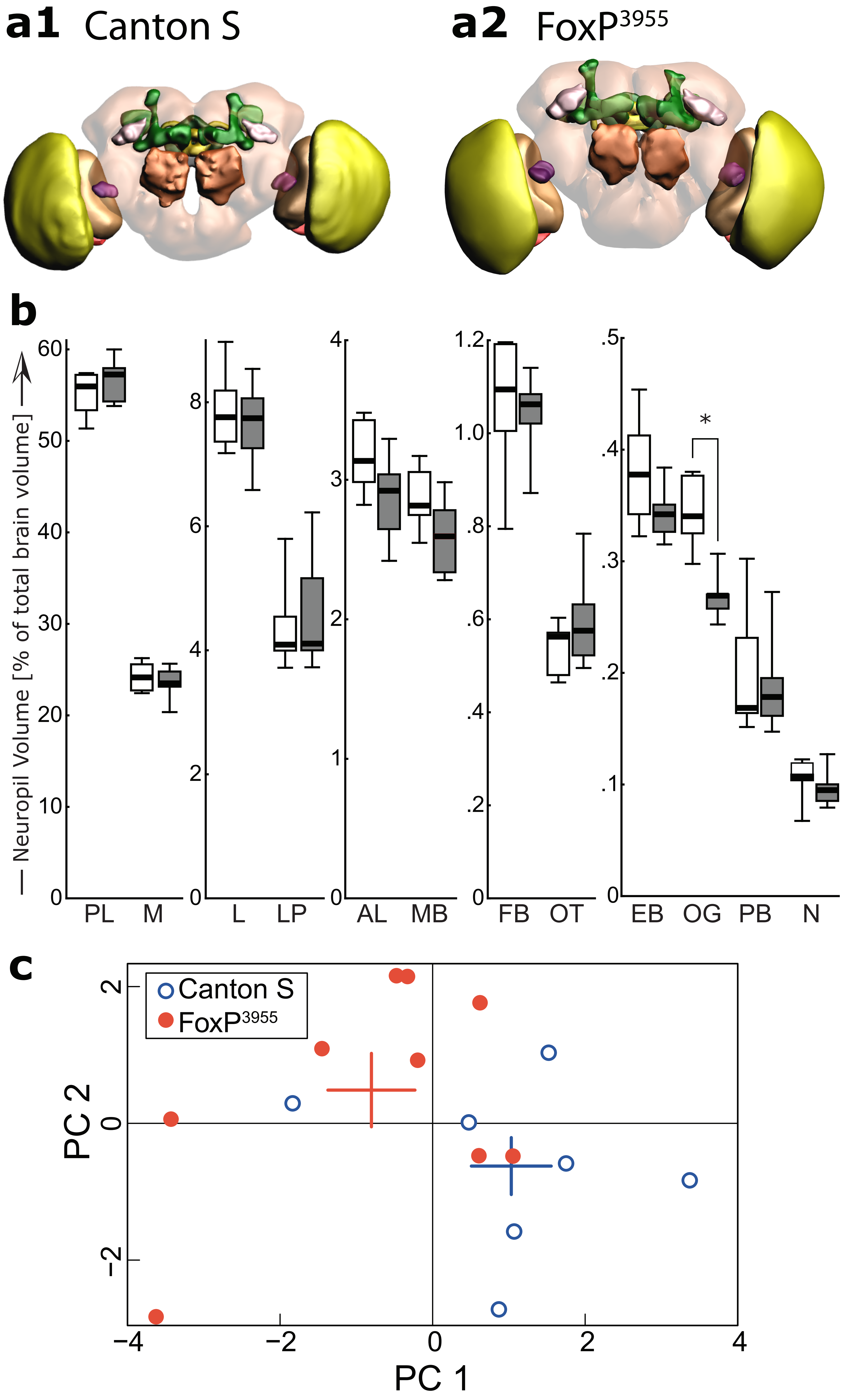

Figure 6: Subtle morphological alterations in the brains of FoxP^{3955} mutants. a, Three-dimensional surface renderings of typical fly brains from wild type Canton S (**a1**) and *FoxP^{3955}* mutants (**a2**). In the online version, clicking on the reconstructions will activate the 3D features of the figure and allow for interactions with the object in space. The different neuropil areas can be selected in the pop-up menu. **b**, Quantitative volumetric analysis of eleven major neuropils (M – medulla, L – lobula, LP – lobula plate, MB – mushroom bodies, AL – antennal lobes, FB – fan-shaped body, OT – optic tubercle, EB – ellipsoid body, OG – optic glomeruli (purple in **a**), PB – protocerebral bridge, N – noduli) revealed a significant reduction in the volume of the optic glomeruli in FoxP^{3955} flies (Mann-Whitney U-Test, U=2.0, $p<0.002$). The volume of the remaining neuropils (denoted PL – protocerebral lobes) did not differ significantly. Asterisk – significant difference with a Bonferroni-corrected level of $p<0.004$. Black stripes – median, boxes – 25-75% percentiles, whiskers – total range. Grey boxes indicate *FoxP^{3955}*, white boxes Canton S. **c**, Principal Components Analysis of the volumetric data. Plotted are the factor loadings of the individual flies on the two first components. Colored bars indicate means and standard errors (PC). Factor loadings are significantly different between Canton S and *Foxp^{3955}* for PC1 (Mann-Whitney U-Test, U=52.0, $p<0.04$), but fail to reach significance for PC2. Number of brains analyzed: 7 (Canton S) and 9 (*Foxp^{3955}*).
